# Interligand Coupling Drives Fast Triplet Energy Transfer Routes in PbS/Tetracene Quantum Dot Hybrids

**DOI:** 10.1021/acsnano.5c16982

**Published:** 2025-11-14

**Authors:** Benjamin Feingold, Nicholas F. Pompetti, Marissa Martinez, Taylor J. Aubry, Jeffrey L. Blackburn, Obadiah G. Reid, Matthew C. Beard, Justin C. Johnson

**Affiliations:** † Chemistry and Nanoscience Center, 53405 National Renewable Energy Laboratory, 15013 Denver West Pkwy, Golden, Colorado 80401, United States; ‡ Department of Chemistry, 1877 University of Colorado, Boulder, Colorado 80305, United States

**Keywords:** quantum dot, ligand, triplet, excimer, photoluminescence

## Abstract

The binding of photoactive organic ligands to inorganic quantum dots (QDs) creates a versatile hybrid architecture that allows access to photophysical processes such as efficient triplet exciton generation with near-infrared radiation. Here we report the subnanosecond generation of a hybrid triplet state with mixed ligand-QD character by replacing native oleate ligands on small PbS QDs with 5,12-tetracenepropiolic acid, a bifunctional ligand with two carboxylic acids that tends to lie face-on with the QD surface at low loadings. The face-on geometry engenders a regime of strong electronic coupling that is evident in steady-state absorption and hastens triplet energy flow by several orders of magnitude compared with more typical tetracene-based ligands exhibiting weak coupling. We further determined via Fourier transform infrared (FTIR) and supported by density functional theory (DFT)-based geometry optimizations that high ligand loading causes a shift in QD-ligand mutual disposition toward an edge-on geometry that instigates the formation of intermolecular excited states characterized by triplet excimer-like features in photoluminescence and transient absorption. Our results demonstrate the ability to control strongly coupled ligand-QD systems toward ultrafast generation of photophysically relevant species such as triplets that are valuable for photon upconversion and catalysis.

## Graphical abstract

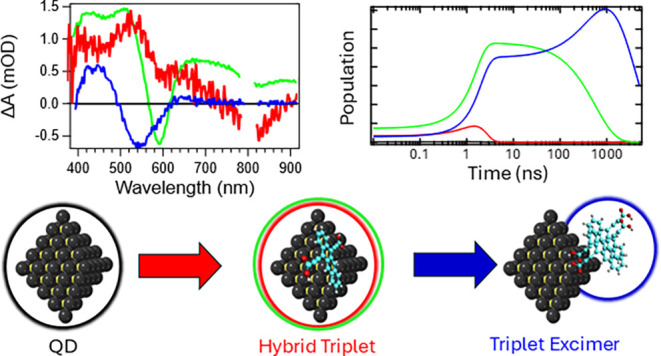

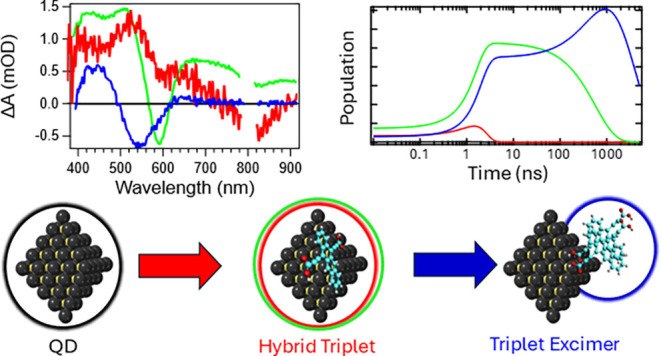

## Introduction

Engineering the ligand shell of semiconductor quantum dots (QDs) enables broad tunability toward functionalities associated with energy conversion and optoelectronics, ranging from photocatalysis to light-emitting diodes.^1–4^ Relatively narrow band gap QDs are ideal for photon upconversion and downconversion applications due to their strong near-infrared absorption and emission and interfacial energy transfer time scales that are typically fast enough to outcompete excited-state decay.^5–10^ Key to the mechanism of these applications is the efficient production of molecular triplet excited states on the organic ligand, which for triplet–triplet annihilation-based upconversion (TTA-UC) involves subsequent annihilation in a bimolecular fashion to generate an emissive singlet on a separate emitter.^11,12^ To date, most studies on QD-ligand triplet transfer systems take place within the weak electronic coupling regime (all instances of coupling refer to electronic states from here onward), wherein the ligand and QD are bound together with mild perturbation of the partner electronic states, but there is no significant hybridization or mixing between the QD and ligand states. It should be noted that despite the absence of hybridization or mixing in the weak coupling regime, the ligand choice can still significantly impact the properties exhibited by the QD-ligand system.^13–17^ This regime is typified by an absorbance spectrum of the combined QD-ligand system that appears roughly as a linear combination of the ligand and QD spectra, with some shifting of the features.^5,6,14^ Within the weak coupling regime, triplet transfer between QD and ligand typically occurs on a time scale of tens to hundreds of nanoseconds for tetracene-based ligands bound to PbS and PbSe QDs, far slower than energy transfer involving singlets and potentially hindering the versatility of TTA-UC schemes.^5,7,8,18^

Compared to the weak coupling regime, there has been relatively little study upon the implications of triplet generation via strong QD-ligand coupling systems. The strong coupling regime is typified by broadening and shifting of the absorbance spectrum such that the combined QD-ligand system cannot be constructed as a linear combination of the ligand and QD spectra.^19–21^ Further, within the strong coupling regime there is significant hybridization of the ligand and QD electronic states. Our group has demonstrated how strong coupling manifests spectroscopically and how the nuanced interplay of 5 nm PbS QDs and 5,12-tetracenepropiolic acid (Tc-DA) ligand geometries, particularly through space, engender strong coupling as evidenced through steady-state absorption spectroscopy.^19^ Wang et al. have reported on a strongly coupled vinyl-anthracene/Si QD system wherein the triplet spectral features are strongly distorted while being generated more quickly than in a related ethyl-anthracene/Si QD system.^20^ By switching from an ethyl to vinyl linker, the study used a covalent “through-bond” connection to enhance the electronic coupling strength. Using these prior studies as precedence, we predict that leveraging similar Tc-DA/PbS geometries as discovered in our previous work, but with smaller QDs (2.7 nm) that allow ligand triplet generation, may enhance interfacial energy transfer rates compared to related systems.^5,6,9,22,23^

Tc-DA was designed to bond to semiconductor surfaces along the short tetracene axis via the carboxylic acid motif (Figure 1A inset, red structure). Its behavior has previously been studied by our group both in solution and as a ligand bound to films of 5 nm diameter (0.9 eV band gap) PbS QDs.^19,24^ Briefly, Tc-DA in dimethylformamide (DMF) solvent was found to exhibit cofacial aggregation that strongly depends on concentration and which enables pathways to potentially useful excited states unavailable to the monomeric species.^24^ When tethered to large PbS QDs, Tc-DA is observed to bind with various tilt angles dependent on the concentration of Tc-DA used for exchange, as inferred from several different spectroscopic techniques (most notably FTIR).^19^ Strong coupling—indicating hybridization of Tc-DA and the QD states—was found to be present to varying degrees across all binding geometries and is visible as broadened, shifted peaks in the absorption spectrum when compared to only Tc-DA in solution. Evidence for altered excited-state dynamics upon strong coupling was observed in an ultrafast relaxation component, but the ligand triplet was energetically inaccessible.

**Figure 1:**
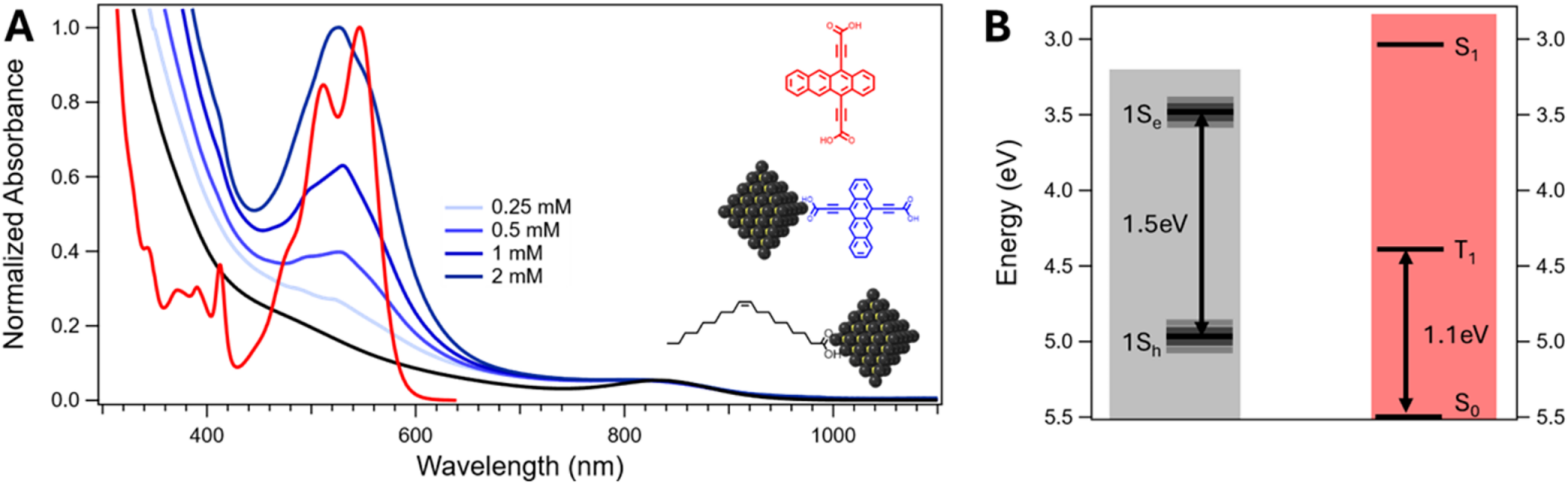
(A) Normalized absorption spectra of PbS/Oleate (black), 1 mM Tc-DA in DMF (red), and PbS/Tc-DA ligand exchanged with varying concentrations of Tc-DA (blue). The inset shows model structures of each species with color coding. (B) Energy level diagram (relative to vacuum) corresponding to hypothetical weakly coupled triplet transfer from PbS QD to Tc-DA.

Employing smaller QDs enables access to different structural geometries, more highly quantized states, and a much higher 1S_h_-1S_e_ (exciton) energy (∼1.5 eV) that lies above the Tc-DA triplet energy (∼1.1 eV).^19,24^ The small QDs also exhibited colloidal stability even after exchange which contrasts with the large PbS QDs that were not colloidally stable after exchange. Colloidal stability allows for minimization of studies on films, which display enhanced light scattering and additional pathways for exciton and/or charge decay.^25^ As a result, here we verify ligand geometries that lead to strong coupling between small PbS QDs and Tc-DA and then evaluate its consequences for dynamics. We find ps-scale triplet transfer that is much faster than observed previously for weakly coupled systems, potentially reducing competition with deleterious pathways. We also find that approaching complete exchange produces triplet states with intermolecular character, a new emergent property for QD-ligand samples.^5,6,18^

## Results and Discussion

### Steady-State Absorbance

The PbS QDs used for this study were synthesized following a synthetic method for producing low polydispersity QDs of varying sizes capped with oleate ligands.^26^ In contrast to our previous study, which utilized a solid-state exchange due to colloidal instability of the 5 nm diameter QDs, we find that smaller QDs retain colloidal stability after solution exchange.^19^ Representative spectra for an oleate-capped QD solution (black) and a concentration series of Tc-DA exchanged QD solutions (blue), along with a spectrum of 1 mM Tc-DA in DMF (red), are shown in Figure 1A (raw data Figure S5). As will be discussed below, films were also prepared via both drop-casting and dip-coating for specific experiments where solvent interference must be avoided.

In the oleate-capped solution (PbS/Oleate), there is a clear exciton absorption peak centered at 828 nm, corresponding to a ∼2.7 nm diameter PbS QD.^27^ The particle size is confirmed by transmission electron microscopy (TEM), which finds an average QD diameter of 2.85 ± 0.28 nm (Figure S3). This size of QD is typically considered to have an octahedral structure with all surfaces being composed of the Pb-rich (111) facet,^28^ although some degree of surface reconstruction is possible.^29^ The broad absorbance extending to higher energy past the exciton arises from a series of quantum-confined PbS states which continuously become more bulk-like at higher energies, eventually leading to an exponential absorbance tail.^13,30^

In the Tc-DA exchanged solution (PbS/Tc-DA), the primary feature is a broad peak centered ∼530 nm, which corresponds to the Tc-DA ligands attached to the QD surface and strengthens in relative intensity with increasing Tc-DA concentration. This ligand peak is substantially broadened and shifted with respect to the solvated ligand spectrum, demonstrating the strong coupling between the QD and Tc-DA that is likely due to a combination of through-bond and through-space interfacial electronic interactions (vida infra). These distortions are significant even when there are very few Tc-DA molecules loaded onto the QD surface (Tables S1 and S2), which can be seen in the spectrum of 0.25 mM exchange. The distortions are reminiscent of those observed when comparing films to solutions for molecules with strong intermolecular coupling.^31^ However, when comparing the PbS/Tc-DA spectra to that of a neat Tc-DA film, we find significant differences (Figure S7). As the concentration of Tc-DA used for exchange increases, there is a subtler broadening and shifting in relative peak intensities (Figure S8), which is likely a result of intermolecular interactions of Tc-DA molecules on the QD surface as loading increases.^32,33^ ^1^H-NMR and UV–vis spectroscopies are used to quantify the number of ligands vs concentration (Tables S1–S5), revealing a trend of <1 Tc-DA ligand per octahedral facet at 0.25 mM ligand loading solution in DMF, to roughly 3–4 ligands per facet for 2 mM loading solution. The QD exciton feature is also broadened, again signifying strong coupling. Finally, there is a broad-band absorbance enhancement (Figure S9) upon exchange of the oleate ligands for Tc-DA that has been previously assigned to coupling between molecular ligand states and broad QD states.^34,35^

Figure 1B shows the energy levels for PbS/Oleate and for Tc-DA in solution.^17,24^ The QD energy levels are presented as bands to represent the broadening associated with inhomogeneous and homogeneous effects typical for similar sizes of PbS QDs.^36^ Importantly, the numerical values given correspond to a system with negligible coupling between QD and ligand. These values are potentially unrealistic even in cases of weak coupling and will likely contain inaccuracies in the position of relevant energy levels for our strongly coupled system. We present these rough energy levels nonetheless as a guide toward expected behavior.

### Vibrational Spectra

The continued broadening of the ligand peaks with increasing Tc-DA loading in the steady state absorbance spectra suggests the enhanced contribution of intermolecular interactions on the QD surface. To further understand how these interactions could arise from the interfacial structure after ligand exchange, we gathered structural information via FTIR. We initially utilized films instead of solutions as DMF absorbs IR strongly and causes saturation and/or outcompetes the signal from the exchanged QDs at many points across the spectrum even with very short pathlengths (Figures S15 and S17). We find that films of PbS/Tc-DA made from small QDs possess nearly identical features when compared to films previously made from large QDs (Figure S14).^19^ We have further confirmed that the loss in oleate-associated peak intensity is a direct result of Tc-DA exchange rather than solvent-assisted stripping of ligands by observing minimal loss of oleate species during DMF soaking of a PbS/oleate film under representative conditions (Figure S16). While the FTIR features are nearly identical between large and small QD films, the ligands on small QDs do not exhibit the same concentration-dependent geometry change as was previously observed for large QDs. Instead, the trends in FTIR intensities suggest small QD PbS/Tc-DA films most closely resemble the face-on geometry found for large QDs.^16,19,37,38^

The alkyne groups on Tc-DA are incisive vibrational reporters as they exist within a narrow wavenumber range in which DMF does not dominate the spectrum and are sensitive to the chemical environment (Figure S18).^39^ This allows for comparison between the behavior of Tc-DA exchanged QDs in film (Figure 2A) and in solution (Figure 2B). Additionally, as Tc-DA has two alkynes—with one being proximal to each carboxylic acid—the relative intensity and position of the alkyne peaks gives insight into how Tc-DA molecules are binding to the QD surface. For instance, if Tc-DA is bound to the QD through both carboxylic acids in a face-on geometry, then both alkynes should experience a very similar environment. In this case, we would expect the alkyne peaks to have similar intensity and appear at close to the same position, which we largely observe in films and rationalize below. Changes in relative intensity and shifting of the alkyne peaks thus represents a state in which the carboxylic acids of Tc-DA are bind, which we observe in solution.

**Figure 2:**
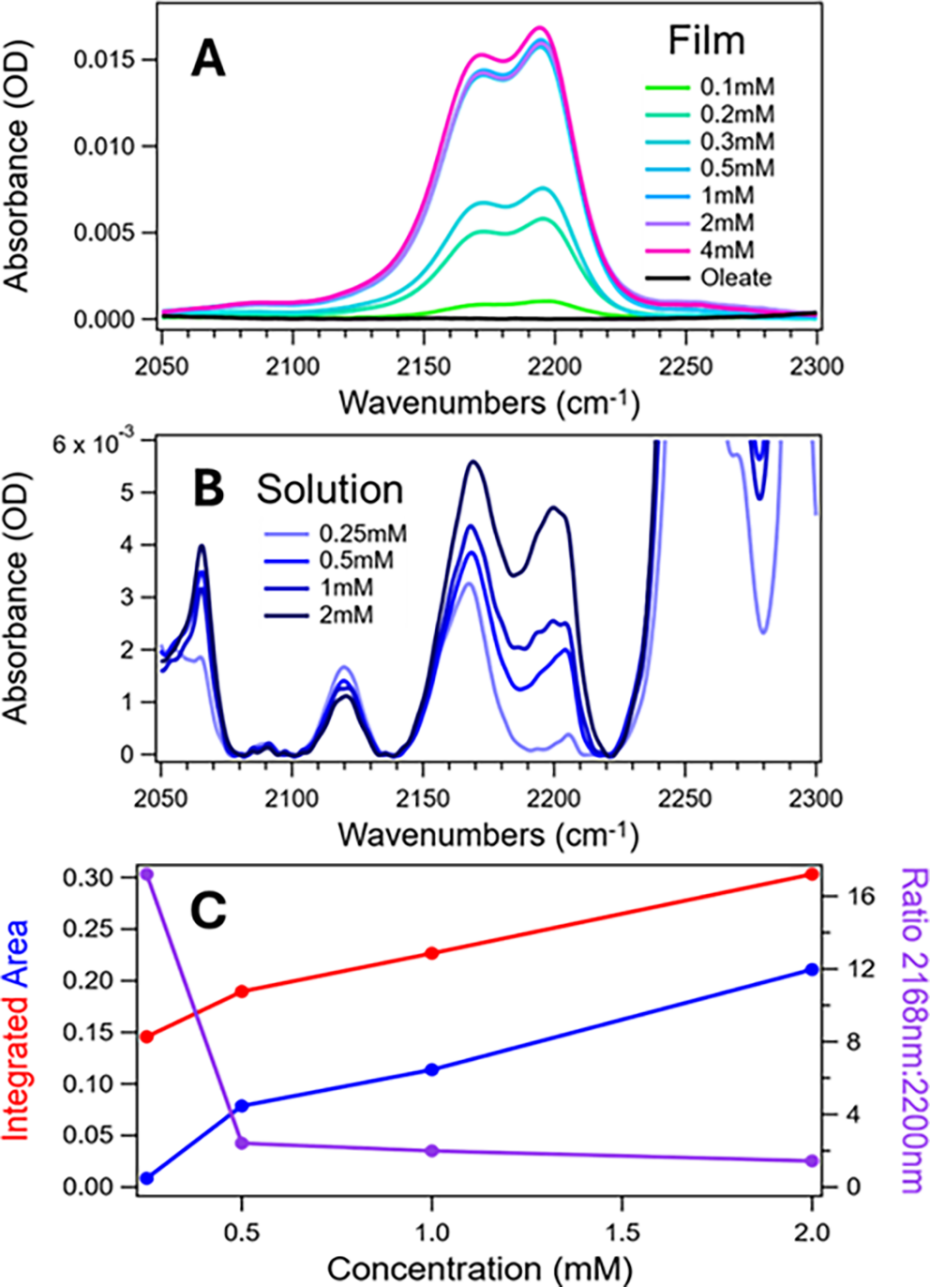
FTIR spectra of the alkyne region for films (A) and solutions (B) of PbS/Oleate and for PbS/Tc-DA with varying concentrations of Tc-DA. (C) Integrated area of the peaks at 2168 cm^–1^ (red circles) and 2200 cm^–1^ (blue circles) for solution spectra. The purple trace shows the ratio between these two peaks as a function of Tc-DA exchange concentration.

For PbS/Tc-DA films (Figure 2A), the alkyne region shows a C≡C doublet, with peaks at 2171 and 2194 cm^–1^. Since oleate has no C≡C and unbound Tc-DA is removed through successive washing, the integrated strength of this band is a proxy for the extent of ligand exchange. We observe a concentration-dependent exchange at low concentrations that saturates above 0.5 mM. We also see no substantive change in the relative intensity between the two peaks as a function of concentration. This contrasts with results on larger PbS QDs, where 0.5 mM shows the greatest intensity for alkyne stretches while higher exchange concentrations have lower intensity, which we assigned to a partial cancelation of oscillator strength by an image dipole that modulates the alkyne stretch oscillator strength as ligands tilt.^19^ The monotonic but highly nonlinear increase in peak strength for small QDs is likely a convolution of two factors: increasing the number of molecules on the surface and a transition to an edge-on geometry as ligands become denser. While the former is an approximately linear factor with concentration, the latter may exhibit a sharper onset and saturation as the number of ligands per facet crosses a threshold. The distinction between this behavior and that of the larger QDs is likely related to steric hindrance that results from the facets of the 2.7 nm QDs being unable to accommodate multiple ligands in a face-on geometry (see Calculations below).

For solutions (Figure 2B), the alkyne region shows a similar C≡C doublet with peaks at 2168 and 2200 cm^–1^. Both peaks also increase in intensity with increasing Tc-DA exchange concentration, representing a greater quantity of Tc-DA loaded onto the QD surface. However, the relative intensities between the two peaks differs starkly from that of films. The peak at 2200 cm^–1^ is initially very weak (0.25 mM) but grows with concentration to become nearly equivalent to the peak at 2168 cm^–1^ (2 mM). The peak at 2200 cm^–1^ also broadens with increasing Tc-DA loading while the peak at 2168 cm^–1^ retains a constant width. The broadening is accompanied by the growth of many different slightly offset peaks (most noticeable in 1 mM) within the Gaussian envelope of the overall band (Figure S19). The presence of multiple peaks and the increasing intensity of those peaks relative to the feature at 2168 cm^–1^ is consistent with a model where ligands assume a face-on geometry with respect to the QD at low Tc-DA loading values but are forced into an edge-on geometry at higher loading values. The single peak at 2168 cm^–1^ represents the alkyne proximal to the carboxylic acid which is bound to the PbS QD. We hypothesize that the additional peaks near 2200 cm^–1^ represent the alkyne extended away from the PbS QD, reflecting slightly different conformations due to interactions with other Tc-DA ligands. The absence of this behavior in films is likely attributable to an increased density of QDs reducing ligand degrees of freedom and the lack of solvent stabilization. These factors may also enforce some degree of symmetry breaking in the bound Tc-DA ligands, resulting in two distinct alkyne peaks even when Tc-DA assumes a face-on geometry.^40^

To elucidate the geometry Tc-DA adopts in solutions of small PbS QDs, we further analyzed the alkyne peak trends in Figure 2B. Figure 2C shows the integrated area of the peaks at 2168 and 2200 cm^–1^ for the solution-exchanged samples as well as the ratio between those values. At low concentrations (0.25 mM), the peak at 2168 cm^–1^ dominates, but increasing exchange concentration brings the two features to near parity. The dramatic shift in ratio between 0.25 and 0.5 mM suggests either a change in the Tc-DA geometry throughout the ligand shell or a substantial adjustment to the relative abundance of (at least) two Tc-DA geometries on the QD surface. As the shift in ratio continues but tapers after 0.5 mM, it is more probable that the shift arises due to the increasing relative abundance of a new geometry rather than a complete replacement of the geometry favored at 0.25 mM. Based on this shift and the results observed from steady state absorbance, we propose that Tc-DA assumes a face-on geometry with respect to the QD surface at low loading values and transitions toward an edge-on geometry with interligand interaction at higher loading values. We further note that the relative intensity of the 2200 cm^–1^ peak is less in 0.25 mM PbS/Tc-DA than when compared to either solutions of only Tc-DA (Figure S19) or for neat Tc-DA powder.^19^ Thus, the very low relative intensity of the 2200 cm^–1^ peak in 0.25 mM PbS/Tc-DA represents exceptionally high symmetry for the Tc-DA molecule.^40^

### Calculated Geometries

To confirm the experimental geometry assignments, DFT-level geometry optimizations (see Methods for details) were performed on 2.37 nm PbS QDs with one, two, or three Tc-DA ligands (Figure 3). 2.37 nm PbS QDs were chosen as they represent the closest size of QD that forms a geometrically “perfect” octahedron comparable to the 2.7 nm QD utilized experimentally. Both the number of ligands and their initialized relative positions on the QD surface affect the resulting geometry. There is a clear trend that as the number of ligands increases, the propensity for Tc-DA to interact intermolecularly increases while QD:ligand interactions decrease.

**Figure 3:**
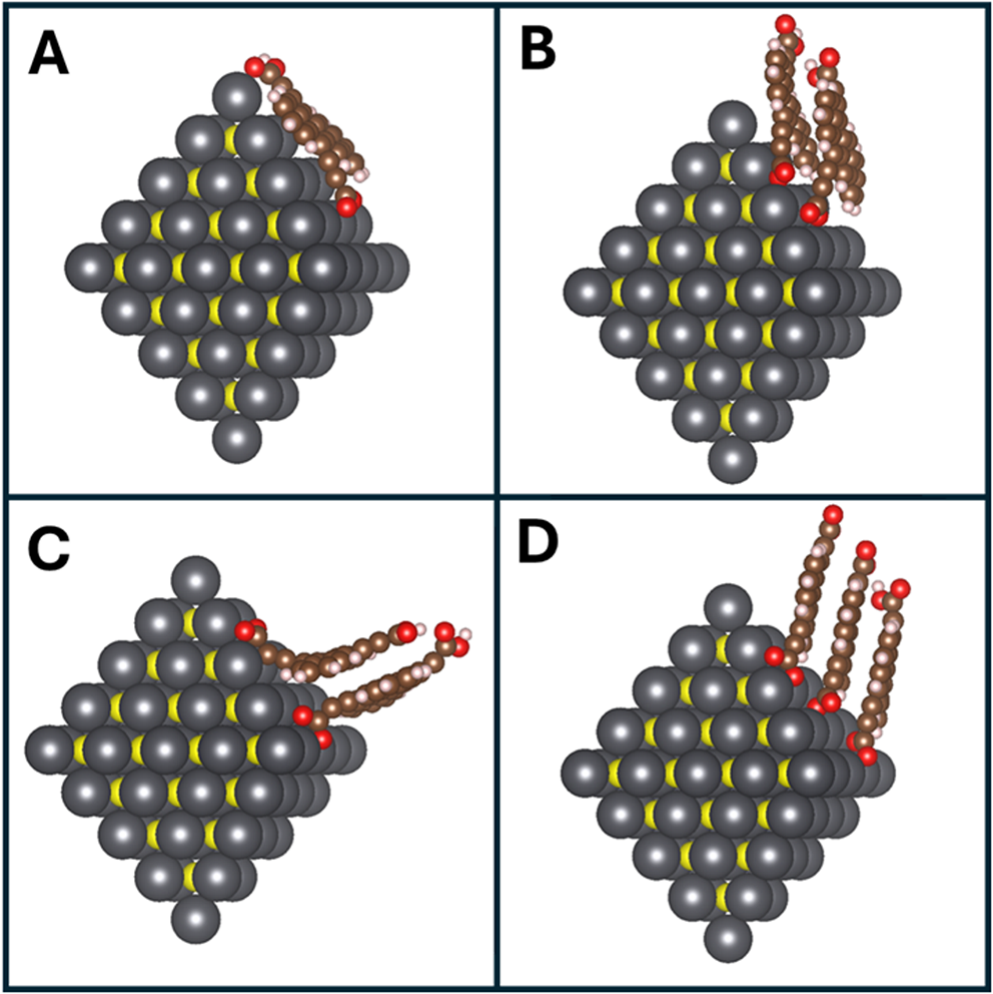
Converged geometry optimizations for 2.37 nm PbS/Tc-DA systems with 1 Tc-DA (A), 2 Tc-DA initialized in parallel (B), 2 Tc-DA initialized orthogonally (C), and 3 Tc-DA (D).

When there is only one Tc-DA (Oleate shown in Figure S20) on the QD surface (Figure 3A), the ligand strongly prefers to lie flat against the surface, with the tetracene backbone parallel to the QD facet. As the number of ligands is increased to two, there is a difference in behavior depending on whether the tetracene backbones are initialized parallel or orthogonal to each other (initial geometries shown in Figure S21). In the parallel case (Figure 3B), the two tetracenes cofacially stack while also orienting to flatten against the QD surface. In the orthogonal case (Figure 3C), the two tetracenes stack cofacially but cannot fully flatten against the QD surface due to their preferred interaction with each other. When the initial geometry inhibits simultaneous optimization for intermolecular and QD:ligand interactions, the intermolecular behavior dominates, driven predominantly by hydrogen bonding interactions. This trend is further illustrated in the three Tc-DA case, where the ligands adopt a cofacial disposition while orienting away from the QD surface. The calculated charge densities for each system (Figures S22 and 23) also reflect this behavior, with the charge density localized on the QD decreasing as the number of Tc-DA ligands increases. Thus, we conclude that face-on ligand geometries are preferred at lower ligand loading while edge-on geometries are preferred at higher ligand loading in agreement with experimental FTIR results. Further, this analysis shows that QD:ligand coupling is reduced as intermolecular interactions become dominant and Tc-DA molecules tilt away from the QD surface.

### Transient Absorption

In order to study how the difference in ligand geometry influences QD exciton evolution upon photoexcitation, we turn to transient absorption spectroscopy (TA), where we selectively excite the QDs with 800 nm pump pulses, eliminating contribution from directly excited ligands. In the weakly coupled regime, we would expect the PbS/Tc-DA spectrum to look similar to the PbS/Oleate spectrum but with a shortened QD-associated species lifetime due to quenching via transfer into the ligand triplet.^5,9,22^ However, the strong coupling regime—suggested by shifting and broadening for PbS/Tc-DA samples in Figure 1A—opens new possibilities that we describe below.

The spectral evolution upon 800 nm excitation for PbS/Oleate and PbS/Tc-DA is shown in Figure 4. For the oleate system, there is a strong bleach associated with the QD exciton centered at ∼800 nm. There is also a broad excited state absorption (ESA) extending from 390 to 750 nm with peaks at 410, 530, and 650 nm. Each band decays with the same lifetime, showcasing that they arise from the same intrinsic QD states. There is an initial decay within the first 20–30 ps that is attributed to Auger recombination.^41,42^ By decreasing pump fluence, this initial decay is significantly reduced and disappears at fluences corresponding to average exciton occupancy ⟨*N*_0_⟩ ≪ 1 (Figure S24), confirming the Auger assignment. After the initial decay, the kinetics flatten, and the signal persists past the 5 ns window with no apparent change in spectral signatures.

**Figure 4:**
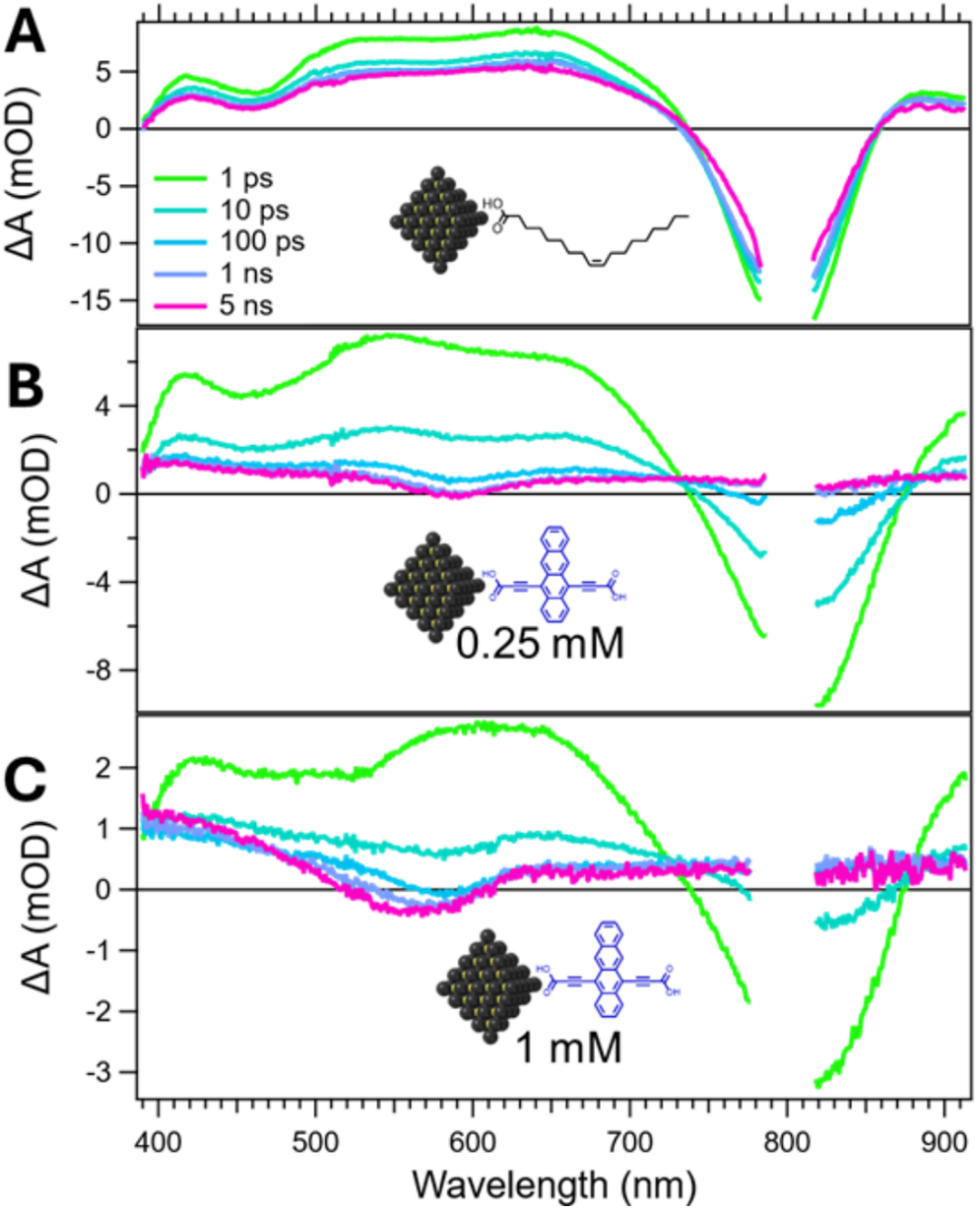
Transient absorption spectral slices from 1 ps to 5 ns for solutions of (A) PbS/Oleate, (B) 0.25 mM PbS/Tc-DA, and (C) 1 mM PbS/Tc-DA after excitation at 800 nm with ⟨*N*_0_⟩ ≈ 0.6.

The spectral evolution upon 800 nm excitation for PbS/Tc-DA differs remarkably from that of the oleate system. A near-infrared exciton bleach is still observed at early pump–probe delays, but it is strongly attenuated, broadened, and slightly shifted—commensurate with what is observed for the exciton via linear absorbance spectroscopy. Concomitantly, there is a broad ESA from 390 to 740 nm with peaks at 410, 550, and 650 nm for the 0.25 mM sample and 410 and 620 nm for the 1 mM sample. The ESA and exciton bleach are simultaneously quenched within ∼1 ns for 0.25 mM and ∼100 ps for 1 mM (Figure 4B,C) to form a new species with a bleach centered at 585 nm. For 0.25 mM PbS/Tc-DA, this feature remains consistent in structure and persists beyond the 5 ns window. For 1 mM PbS/Tc-DA, there is a progressive broadening of the bleach band toward shorter wavelengths, eventually resulting in a negative feature extending from 530 to 600 nm that persists beyond the 5 ns window. Broad ESAs extend to both higher and lower wavelengths relative to these emergent bleach peaks.

In the weak coupling regime, this spectral transition is typically associated with direct triplet transfer from the QD into the ligand.^5,7,8,18^ However, the state which emerges in the strongly coupled PbS/Tc-DA system does not resemble the well-resolved vibronic features of a tetracenic triplet.^24,43,44^ Despite this, the bleach features are aligned in wavelength and broadness with the absorbance features observed for PbS/Tc-DA (Figure 1A), suggesting that the emergent state does possess ligand character. Previous work on strongly coupling QD/ligand systems has shown similar loss of vibronic character in the triplet state.^20^ This nonmolecular triplet was assigned to spatial delocalization of the ligand triplet state over both the QD and ligand. Based on these similarities we propose that the features which grow in during the first 100 ps reflect a hybrid triplet state with character originating from both Tc-DA and the PbS QD surface. The differences between 0.25 and 1 mM PbS/Tc-DA further reflect how the geometry of Tc-DA on the QD surface influences the formation and evolution of the hybrid triplet. We will discuss the nature of this triplet state below.

Since the hybrid triplet feature persists past the time window of the ultrafast TA experiment, we also collected nanosecond to microsecond data via an electronically delayed TA experiment (Figure 5). While PbS/Oleate shows no further spectral changes on a ns-μs time scale, relative to the fs-ns time scale (Figures S28 and S29), the PbS/Tc-DA systems continue to evolve at longer delays. Most notably, the visible bleach feature which arises as the near-IR QD exciton bleach is quenched continues to blue-shift over a ∼100 ns time scale for the 1 mM PbS/Tc-DA sample while not substantially shifting for the 0.25 mM sample over the same time scale. The 0.25 mM PbS/Tc-DA bleach eventually begins to blueshift after ∼500 ns, but not to the same extent as 1 mM. Additionally, the species lifetimes are greatly altered by the exchange. For PbS/Oleate the lifetime is ∼10 μs (Figures S28 and S29) whereas 1 mM PbS/Tc-DA solutions have an ∼5 μs lifetime. The persistence of the hybrid triplet state for several microseconds past when the QD exciton is fully quenched reinforces that the long-lived species in PbS/Tc-DA has little to no core exciton contribution. However, this does not necessarily rule out the possibility of QD involvement via surface states.^7,9^

**Figure 5:**
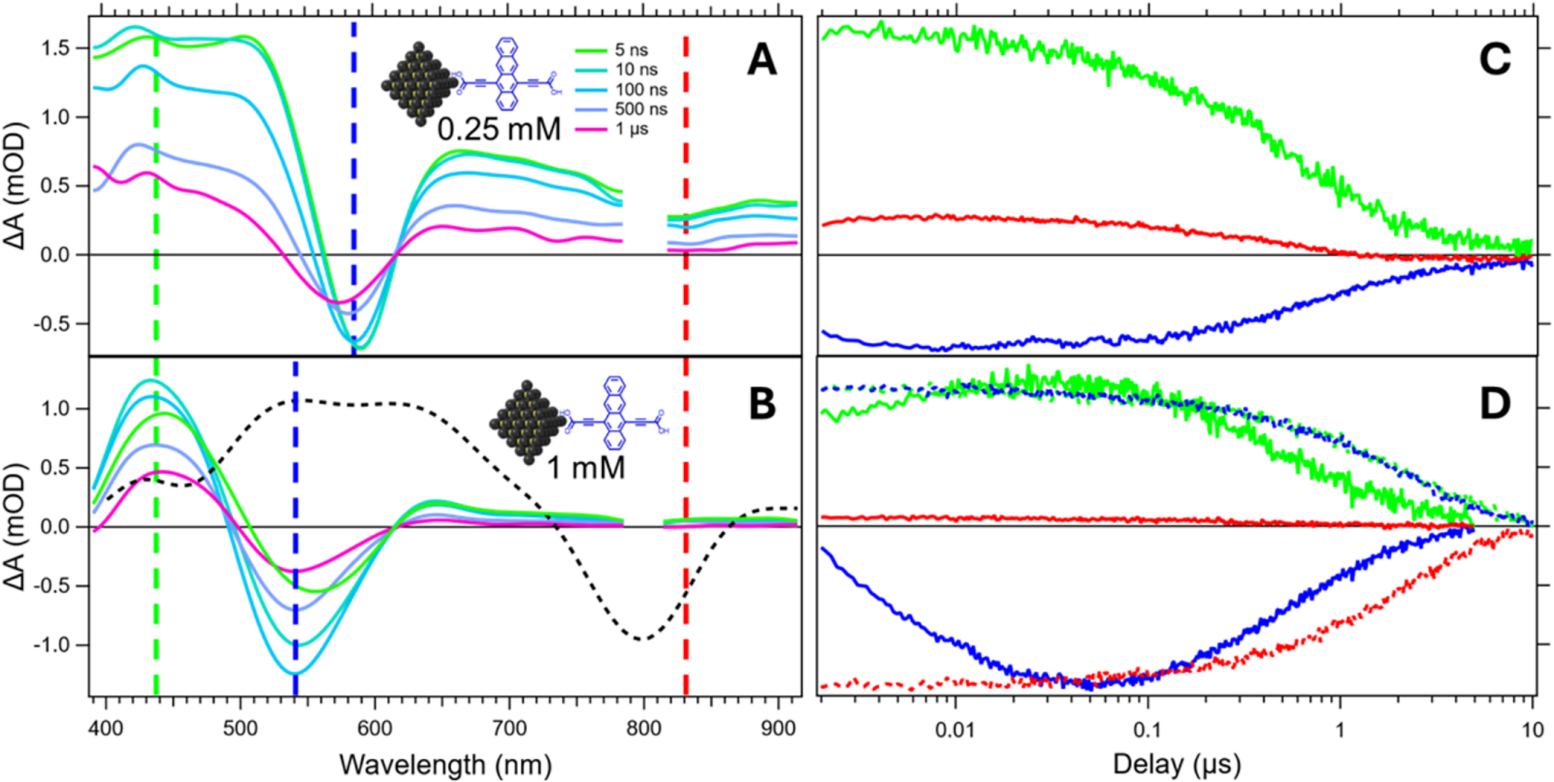
Transient absorption spectral slices from 5 ns to 1 μs for a solution of (A) 0.25 mM PbS/Tc-DA and (B) 1 mM PbS/Tc-DA. Dashed gray line is a normalized spectral slice from a solution PbS/Oleate at 100 ns for comparison. Kinetic slices through QD exciton (820 nm, red), triplet-feature (540 nm, 590 nm; 1 mM, 0.25 mM, blue), and ESA (480 nm, green) for (C) 0.25 mM PbS/Tc-DA and (D) 1 mM PbS/Tc-DA. Dashed lines show the normalized kinetics for a solution of PbS/Oleate at the same wavelengths. Samples were excited at 800 nm with ⟨*N*_0_⟩ ≈ 1.22.

Interestingly, despite the quenching of the QD exciton on a subnanosecond time scale (Figure 4C), the maximum intensity of the hybrid triplet species occurs at ∼100 ns (Figure 5A) for the 1 mM PbS/Tc-DA species. This delayed maximum suggests that there is an intermediate species between the initial excitation of the QD and the final broad state for 1 mM PbS/Tc-DA that is absent for 0.25 mM PbS/Tc-DA. As the QD exciton is fully quenched well in advance of 100 ns, it is unlikely that the continued growth is attributable to ongoing transfer from a purely QD-centered species into the hybrid triplet. Instead, it is more probable that the continued growth arises from changes to Tc-DA orientation and/or electronic state on the QD surface. For instance, the formation of an intermolecular state would result in increasing bleach magnitude as the delocalization of the initial excitation results in (at least) two depleted Tc-DA ground states. We note that a triplet excimer (excited dimer) is a known intermolecular state in organic systems with heavy atoms, and it is energetically accessible here.^45–48^

### Photoluminescence

To test the hypothesis of triplet excimer formation, we collected photoluminescence (PL) spectra of QDs with varying Tc-DA exchange concentrations (Figure 6). The triplet energy level for neat Tc-DA has previously been determined as ∼1.1 eV.^24^ Therefore, if a Tc-DA triplet excimer is forming, we would expect to observe a broad, featureless peak at slightly lower than 1.1 eV, in analogy with singlet excimers at higher energies reported in tetracene derivatives.^49,50^

**Figure 6:**
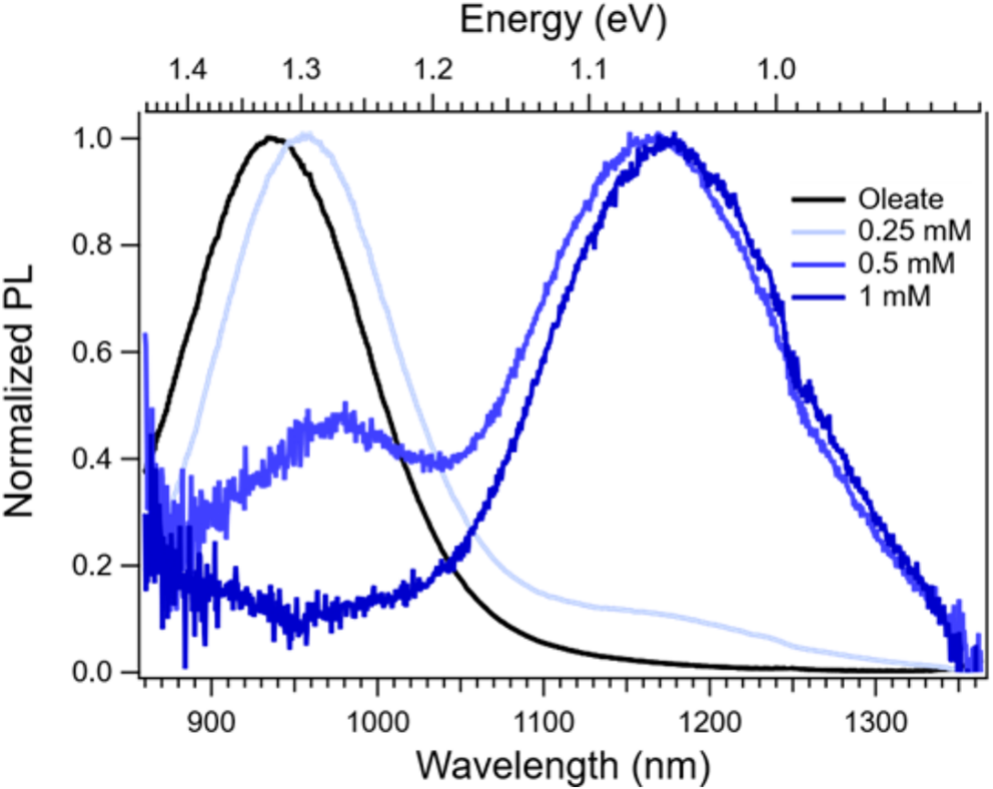
Normalized PL spectra of PbS/Oleate (black) and PbS/Tc-DA ligand exchanged with varying concentrations of Tc-DA (blue). To match the PL intensity of the PbS/Oleate spectrum, 0.25 mM PbS/Tc-DA was multiplied by 610 while 0.5 and 1 mM PbS/Tc-DA were multiplied by 10,200.

For PbS/Oleate, there is a single, very intense PL peak centered at 937 nm which corresponds to emission from the QD exciton. The large Stokes shift observed (0.174 eV) is typical for small PbS QDs.^51–53^ Upon exchange with 0.25 mM Tc-DA the exciton PL peak is shifted to 958 nm and its intensity is substantially quenched, which is consistent with previously shown spectroscopic results (Figures 1 and 4). A clear shoulder also emerges at ∼1180 nm. As the exchange concentration is increased, the exciton feature is further quenched (0.5 mM) and eventually disappears (1 mM) while the shoulder further develops with increasing exchange concentration into an extremely weak, broad feature centered at 1177 nm (∼1.05 eV). This new feature is consistent with the hypothesized formation of a Tc-DA triplet excimer, as it lacks the structure typical of localized-triplet phosphorescence and peaks below the expected triplet energy.^54^ The increasing relative intensity of the triplet excimer with higher Tc-DA concentration also agrees with the proposed shift to an edge-on Tc-DA geometry with intermolecular behavior at high loading. The absence of any structured phosphorescence may result from the proposed hybrid nature of the triplet, which could allow for nonradiative decay through the QD surface states. We attribute the residual QD exciton PL in the 0.25 and 0.5 mM PbS/Tc-DA PL to a minority population of unexchanged QDs. The exciton feature has approximately 10000 times more emission intensity than the triplet excimer feature (Figure S30), so a very small population of unexchanged QDs will appear dominant in PL. Conversely, exchanged and unexchanged QDs should have roughly comparable intensity in TA. The dominance of the exchanged species spectrally and kinetically in TA (Figure 4B,C) again suggests that unexchanged QDs are a minority species.

### Fluence-Dependent Transient Absorption

To rule out assignment to other species previously implicated in QD/ligand samples, we compare the spectral features of the emergent state against known spectra of the Tc-DA cation and anion (Figure S31).^24^ While the broadness of the putative hybrid state’s spectral features make it difficult to precisely rule out any of these contributions, the absence of several characteristic peaks suggests that the PbS/Tc-DA system is distinct from any of them. For both the cation and anion, there are distinctive peaks centered at 350 and 900 nm (Figure S31) which are not present in the hybrid spectrum. Further, the QD exciton has already been fully quenched on the time scale where the ligand-based features become dominant. For a charge-separated system, we would expect there to be a charge on both Tc-DA and in the QD core, and thus for the QD bleach feature to be present. However, previous studies have found that triplet generation can be mediated by QD surface states, which could explain why the exciton feature is absent.^7–9^ To test for the presence of mediating QD surface states, we performed TA measurements across a range of fluences (Figure 7). Assuming surface states are present and influential, as observed in PbS/pentacene samples, increasing fluence should eventually result in saturation of those states. Once the surface states are saturated, higher fluence should no longer result in increased TA signal.^7^ Conversely, if the surface states are functioning as traps then their saturation should lead to faster long-lived state formation.

**Figure 7:**
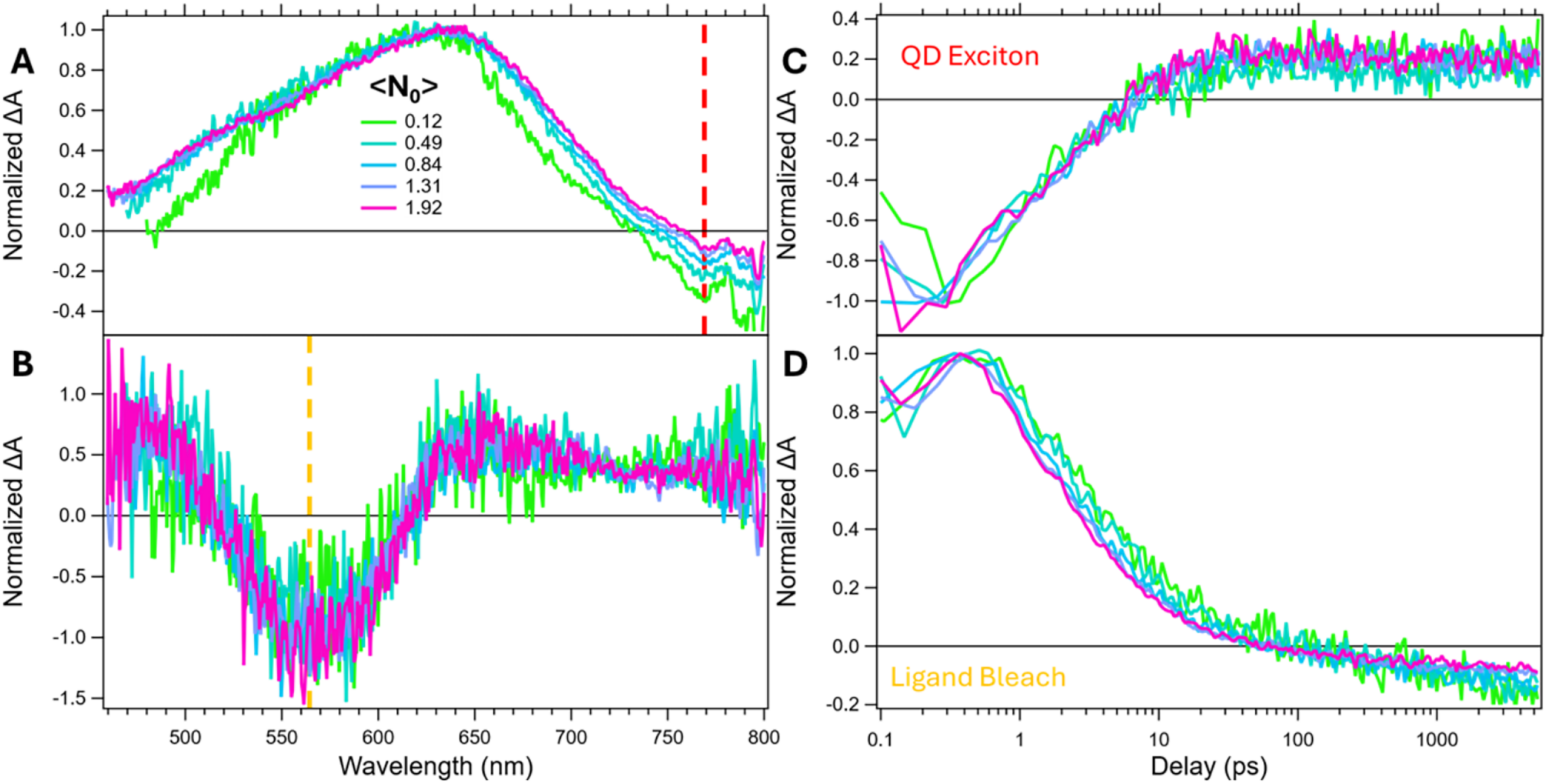
Fluence series transient absorption normalized spectral slices at 1 ps (A) and 5 ns (B) for a solution of 1 mM PbS/Tc-DA. Kinetic slices through the QD exciton (C) and ligand bleach (D). The legend in (A) details the calculated ⟨*N*_0_⟩ for each scan in units of excitons per QD.

We find that the saturation of surface-state population found in PbS/pentacene samples with increased fluence is absent here (Figure S32).^7–9^ Instead, the spectral features associated with the intermediate and long-lived ligand species are unaffected by fluence. Additionally, the QD exciton decay time and the long-lived ligand species rise time remain constant (0.25 mM PbS/Tc-DA fluence kinetics shown in Figure S25). The absence of fluence dependence and the spectral mismatch between the hybrid triplet and the Tc-DA anion/cation strongly suggests that neither anion/cation nor surface states are contributing to the observed dynamics and spectra. We further note that this behavior strongly deviates from typical Auger-mediated QD exciton decay demonstrated in the power dependence of oleate-capped PbS (Figure S24).

The Tc-DA triplet was also studied in isolation via sensitization (Figure S33). In isolated solution-phase Tc-DA molecules, there is a strong ESA centered at 380 nm which is not obviously present in the PbS/Tc-DA spectra, although it is difficult to probe this region in QD/Tc-DA samples because of the parasitic absorption of UV probe light by PbS.^24^ However, there is a broad, strong ESA from 400 to 530 (550) nm in the 1 mM (0.25 mM) PbS/Tc-DA spectrum that is similar to the Tc-DA triplet sensitization. The sensitized Tc-DA triplet has no significant spectral signatures to the red of ∼650 nm, whereas the transient features of the PbS/Tc-DA samples exhibit a broad ESA extending beyond 620 nm and well into the NIR (Figure S34). While some mismatch between the observed spectral features for the PbS/Tc-DA system species and that of Tc-DA alone are evident, the spectra also gain similarity to the TA features of PbS/Oleate, including the broad ESA extending into the NIR (Figure S35). Overall, the spectra of PbS/Tc-DA differ substantially from that of the PbS/oleate samples even at single picosecond time scales (Figure 4). Based on the absence of narrow Tc-DA features and the addition of broad QD features, we again assert that the long-lived species is a hybrid triplet formed via wave function overlap of Tc-DA and the PbS QD.

### TA Global Fitting

Through fitting of the ns-μs TA data in Figure 5, we derive species-associated spectra that represent the dominant long-lived species in PbS/Tc-DA (Figure 8A–C). Excitation of the QD generates a species reminiscent of the PbS/Oleate exciton (black). This species is rapidly quenched to form two hybrid QD:Tc-DA triplets, with one having more tetracenic character (red) and the other more mixed QD-ligand character (green). Some QD character remains in the tetracenic triplet species, and it does not exactly match the literature tetracene triplet, so it is likely hybridized to some extent.^5,22^ Both triplet species then proceed into the triplet excimer (blue) final state at different rates. Based on this analysis, we propose that PbS/Tc-DA follows a bifurcated kinetic scheme (eqs 1 and 2), where *X*_QD_ is the initially excited QD, T_tet_ is the tetracenic triplet, T_hyb_ is the hybrid triplet, T_exc_ is the triplet excimer, and GS is the ground state.

1a $$X_{\mathrm{QD}}\to T_{\mathrm{tet}}\to T_{\mathrm{exc}}\to \mathrm{GS}$$

1b $$X_{\mathrm{QD}}\to T_{\mathrm{hyb}}\to T_{\mathrm{exc}}\to \mathrm{GS}$$

This kinetic scheme is indicated graphically in Figure 8D. We find that the fs-ns and ns-μs TA data for both 0.25 and 1 mM PbS/Tc-DA can be readily fit with this kinetic scheme, which is described in more detail in the SI. Schemes lacking the intermediate triplet failed to reproduce the data, which can be detected by monitoring the delayed emergence of the 585 and 545 nm bleaches in 1 mM samples leading to sigmoidal structured kinetics (Figure S27). A model involving the sequential population of T_tet_ and T_hyb_ species was also attempted, but fits were clearly inferior (Figures S40, and S41).

**Figure 8:**
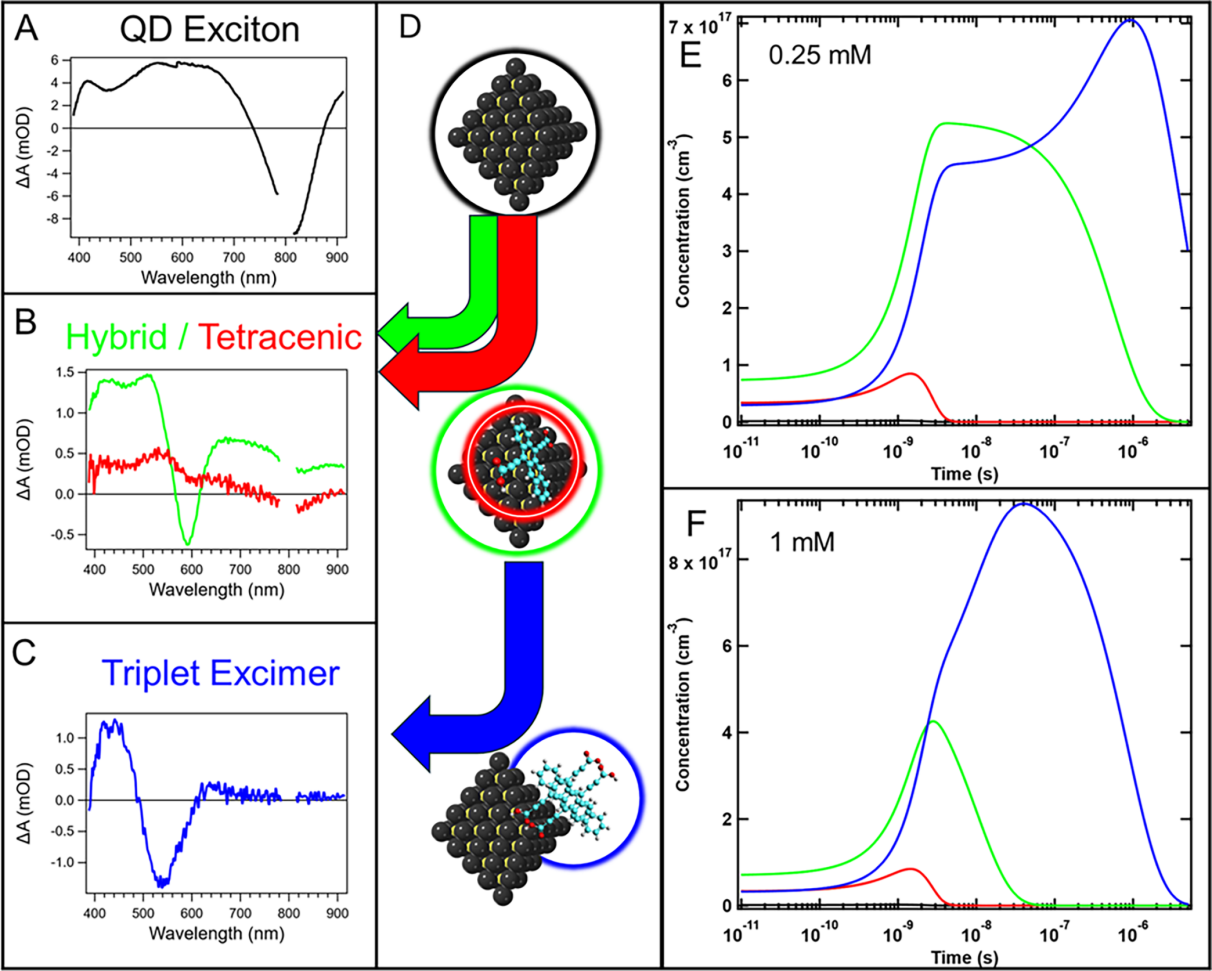
(A–C) Species-associated spectra and (D) graphic representation of the geometry for initial QD excitation, tetracenic triplet, hybrid triplet, and triplet excimer. Species populations of fits using solved differential equations for (E) 0.25 and (F) 1 mM PbS/Tc-DA. Pb atoms are shown in black, S atoms in yellow, H atoms in white, and O atoms in red. Tc-DA carbon atoms are shown in cyan for contrast.

The hybrid triplet proceeds into T_exc_ at a slower rate compared to T_tet_, and the rate constant differs greatly between 0.25 and 1 mM PbS/Tc-DA (Figure 8E,F). The population profiles show the relative contributions of each species at various times, demonstrating how higher surface coverage at 1 mM favors species associated with edge-on and aggregate geometries at earlier times, opposite to that of 0.25 mM. We attribute the slower rate of excimer formation at low concentration to the inherent barrier for localization of T_hyb_ to a specific QD surface position where an aggregated Tc-DA geometry is present for T_exc_ formation. The more than 10-fold difference in rate between 0.25 and 1 mM is then attributable to the vastly decreased density of aggregated Tc-DA species on 0.25 mM PbS/Tc-DA. For 1 mM PbS/Tc-DA, aggregated Tc-DA is the dominant species and thus the rate is much faster. The raw TA data reveals evidence for the distinction in rates (Figures 4 and 5), where 0.25 mM PbS/Tc-DA shows only minor shifting of the bleach from 585 nm, whereas 1 mM PbS/Tc-DA shows rapid growth of additional bleach at 545 nm, which then becomes the most intense segment of a broad bleach. This difference in rate also explains why the visible bleach at 545 nm in 1 mM PbS/Tc-DA appears to continue growing in intensity out to nearly 100 ns whereas the visible bleach at 585 nm in 0.25 mM PbS/Tc-DA reaches its apparent maximum after only ∼ 5 ns. At even later times (several μs), the 545 nm feature grows in for 0.25 mM PbS/Tc-DA and becomes dominant, further agreeing with a slower rate of transfer. The ongoing emergence of these bleaches past when the initial QD excitation is fully quenched also validates the notion of an intermolecular triplet excimer state wherein ground state depletion for two Tc-DA molecules results from one initial excitation.

The tetracenic triplet rapidly transfers into T_exc_ and is likely formed in edge-on ligand geometries, leading to weaker coupling with the QD surface (scheme in Figure 8D). The weaker coupling then results in the increased tetracene-like character observed for this species. We conclude that this triplet can rapidly transfer into the triplet excimer state due to the accompanying π-stacking interactions that are exclusively found in aggregated Tc-DA edge-on binding geometries.

## Conclusions

We have investigated the structural, spectral, and dynamic changes that occur when exchanging passivating oleate ligands on small (2.7 nm diameter) PbS QDs with tetracene molecules functionalized with two carboxylic acids. We find that Tc-DA strongly couples to PbS QDs and at high loading forms intermolecular species on the QD surface. The strong coupling between QD and Tc-DA results in the formation of a hybrid triplet with mixed character on a hundreds of picosecond time scale after excitation of the QD. This formation is 2–3 orders of magnitude faster than what is typically reported for weakly coupled PbS­(e)/ligand or for strongly coupled Si/ligand systems under low-fluence excitation conditions.^5–9,20^ The faster formation may be attributable to the diacid functionalization of Tc-DA that encourages face-on geometries toward the QD surface. The increased proximity between ligand and QD likely increases spin–orbit coupling and hastens the sub-ns development of triplet character.

Finally, we show that the ultimate photophysical outcomes can be directed by controlling the geometry and packing density of photoactive ligands on QD surfaces. We found that Tc-DA geometry shifts from predominantly face-on to edge-on with respect to the QD surface with increased Tc-DA loading and that these ligands are then capable of forming a triplet excimer, a previously unreported species for NIR QD-ligand systems that is consistent with our observation of both a new emissive state with very large Stokes shift and a distinct transient absorbance signature. Whereas in similar systems outcomes of processes like upconversion are reported vs ligand type and QD size, the actual ligand geometries from detailed structural data have not been specifically correlated to excited-state behavior. Our combined structural and photophysical investigation highlights the importance of understanding ligand geometry as a method through which other dynamic and spectroscopic results may be interpreted, including intermolecular phenomena on QD surfaces.

## Materials and Methods

### Computational Methods

#### Calculation of Predicted Geometries

All density functional theory (DFT) calculations were performed with ORCA (version 5.0.3).^55,56^ Geometry optimizations were performed using the BP86 exchange-correlation functional, a generalized gradient approximation functional chosen for its performance in predicting experimental bond distances and vibrational frequencies in various transition-metal complexes, while remaining computationally efficient for the larger hybrid quantum dot-organic systems studied here.^57–60^ To account for dispersion interactions, Grimme’s D3 correction with Becke-Johnson damping (D3BJ) was included.^61,62^ A split-valence def2-SVP basis set was employed for all atoms and the associated def2 effective core potential was used to treat relativistic core electrons efficiently in for Pb atoms.^63^ The resolution-of-identity approximation with the RIJCOSX method and def2/J auxiliary basis set were employed to accelerate the calculations.^64,65^ Convergence was enforced using the DIIS algorithm with tightscf and slowconv settings.^66,67^

For geometry optimizations, the PbS quantum dot core was held fixed by applying constraints to the Pb and S atoms, while ligands bound to the surface were allowed to relax fully from initial binding configurations.

Charge density difference images were created by subtracting the charge density of the 2.37 nm PbS QD from the charge density of the various ligated PbS QD systems and are displayed at the 1 × 10^–4^ e/Å^3^. Charge density integration was performed directly from the cube files generated in ORCA. The electron density values stored on the real-space grid (in e/Bohr^3^) were summed over assigned regions, with the volume of each voxel computed from the cube lattice vectors. Each grid point was assigned to the nearest atom if it fell within its van der Waals radius. If the point was within the radii of multiple atoms, it was assigned to the closest one. Because cube files represent only the density included in the chosen basis and pseudopotentials, and because of discretization on a finite grid, the integrated electron counts do not exactly reproduce the total number of electrons reported in the SCF calculation. However, the relative trends between systems are robust as the quantum dot geometry is not changing and form the basis for our analysis.

## Experimental Methods

### PbS QD Synthesis

Lead oxide (99.9995%, Puratronic) was purchased from Alfa Aesar. Oleic acid (90%), octane (anhydrous, ≥99%), acetonitrile (>99.9%), trifluoroacetic acid (99%), trifluoroacetic anhydride (≥99%), triethylamine (≥99.5%), toluene (≥99.5%), methyl acetate (anhydrous, 99.5%), tetrachloroethylene (anhydrous, ≥99%), deuterated chloroform (anhydrous, ≥99.8%), ferrocene (98%), 2-isopropylaniline (97%), 3,5-Bis­(trifluoromethyl)­phenyl isothiocyanate (98%) were purchased from Sigma-Aldrich. Standard grade, uncoated, 2 mm calcium fluoride IR windows were purchased from Knight Optical.

PbS QDs were synthesized under oxygen- and water-free conditions using a synthesis developed by Hendricks et al. For the synthesis of 2.7 nm PbS/Oleate, 2.9 g of Pb­(oleate)_2_ was added to 36.875 mL of octane in a 100 mL three-neck flask with an air-free valve. In a 20 mL scintillation vial, 1.016 g of *1-[3,5-Bis­(trifluoromethyl)­phenyl]-3-(2-propan-2-ylphenyl)­thiourea* was added with 1.25 mL of diglyme. Both solutions were brought to 90 °C for 10 min with stirring and under a N_2_ flow. During this time, the respective solids dissolved, and then the entire thiourea solution was rapidly injected into the Pb­(oleate)_2_ solution. The reaction was removed from heat after ∼ 60 s, and the product was cooled to RT and dried under vacuum for >1 h. Once dried, the flask and contents were transferred to a nitrogen-filled glovebox, dissolved in a minimal volume of toluene, and centrifuged at 7000 rpm for 10 min, followed by 4–6 cycles of precipitation/centrifugation with toluene (solvent) and methyl acetate (antisolvent). After 4–6 cycles, the product was resuspended in hexane and stored in a nitrogen-filled glovebox.

### Thiourea Synthesis

*1-[3,5-Bis­(trifluoromethyl)­phenyl]-3-(2-propan-2-ylphenyl)­thiourea* was synthesized by dissolving 5.397 g of 3,5-Bis­(trifluoromethyl)­phenyl isothiocyanate in 20 mL toluene and mixing with 2.6974 g of 2-isopropylaniline in another 20 mL of toluene in an Erlenmeyer flask. After the mixture was stirred for several minutes, the product was rotovapped to remove toluene. The resulting product was a fluffy orange-pink powder. To purify the product, the powder was recrystallized by adding just enough heated toluene to dissolve. A 10:1 mixture of hexane:DCM was then added to promote recrystallization. The flask was covered and placed in a freezer overnight to finish recrystallization. The resulting product was vacuum filtered and rinsed with hexane then allowed to dry under vacuum filtration overnight. After drying, 6.691 g of *1-[3,5-Bis­(trifluoromethyl)­phenyl]-3-(2-propan-2-ylphenyl)­thiourea* was recovered (82.7% yield).

### 5,12-Tetracenepropiolic Acid Synthesis

See JACS Pompetti et al. 2024 for full details of synthesis.^24^

### 5,12-Tetracenepropiolic Acid Solution

Solutions were prepared by weighing out 1–2 mg of powder via an analytical balance. A volume of DMF was then calculated and added to the powder, which readily dissolves into a bright red solution. Aliquots of the stock solution were diluted with further DMF to produce lower concentration solutions.

### Solution Ligand Exchange

Twenty-five μL of approximately 25 mg/mL PbS stock solution in hexane was transferred into a glass one dram vial. The hexane was allowed to evaporate and then the dry QDs were readily resuspended in 500 μL of TCE. 500 μL of Tc-DA solution in DMF was added to the dram vial and the mixture was left to exchange with stirring for 1 h. 0.5, 1, and 2 mM exchanges were stable in the 50–50 DMF-TCE mixture but were centrifuged and resuspended in 50–50 DMF-TCE to minimize unbound ligands. 0.25 mM exchange was not stable in the solvent mixture and had to be centrifuged and resuspended in TCE. Centrifugation did not result in significant changes to the absorbance spectra of PbS/Tc-DA samples (Figure S10). Notably, adding 500 μL of neat DMF to the QD-TCE solution resulted in precipitation of the PbS QDs within 20 min regardless of stirring. This suggests that the ligand exchange confers stability in DMF. All steps were performed in an N_2_ glovebox.

### Film Ligand Exchange

The PbS/Oleate films were placed into a 5 mL beaker with about 500 μL of Tc-DA in DMF solution and allowed to exchange for an hour. The films were then removed from the beaker, washed in DMF to remove excess free ligand, and then washed in hexane to promote drying.

### Dip-Coated Films

Cleaned CaF_2_ substrates were introduced into a N_2_ glovebox for preparing films for FTIR. About 500 μL of 5 mg/mL PbS QD in hexane solution was deposited into a 5 mL beaker. The beaker size was chosen purely to minimize the required volume of solution for dip-coating. Separately, about 500 μL of Tc-DA in DMF solution was deposited into a 5 mL beaker. The films were submerged into the beaker of QD solution using tweezers and then pulled out after a few seconds. Excess solution was knocked back into the beaker. The films were allowed to dry and then submerged into the beaker of Tc-DA solution. The films were allowed to exchange for an hour before being removed from the beaker. The films were then washed in DMF to remove excess free ligands and then washed in hexane to promote drying. After the films were dry, the dip-coating procedure was repeated 2–4 more times to build up layers of exchanged QD.

### Spectroscopic Methods

NMR data was collected using a Bruker AS400 instrument. Samples were prepared according to solution ligand exchange procedure but were resuspended in CDCl_3_ instead of TCE or DMF. Experiments were performed using a 90° magnetization to maximize signal and attain as analytical a result as possible. A 100 second relaxation delay was used as the slow tumbling of the QDs resulted in signals that persist significantly beyond the standard NMR relaxation delay.UV/vis/NIR absorption was measured on a Cary 5000 instrument. FTIR spectra were collected using a Nicolet iS50R FT-IR instrument in transmission mode. Film data were collected by casting PbS/Tc-DA onto small CaF_2_ substrates following the dip-coating procedure. Solution data was collected by injecting PbS/Tc-DA into a demountable liquid cell with 500 μm pathlength. Solutions were prepared following the solution exchange procedure, but all samples were resuspended in TCE to minimize DMF interference. Photoluminescence spectra were collected with a Princeton spectrometer. Photoluminescence spectra were collected using a custom-built Princeton Instruments spectrometer. A 1D liquid-nitrogen-cooled InGaAs array (PyLoN-IR) was used for SWIR measurements (850–1550 nm). SWIR spectra were calibrated using a SWIR quartz tungsten halogen lamp from Princeton Instruments. Dual monochromators (HRS 500) were used to achieve pseudomonochromatic excitation from an Energetiq EQ99x laser-driven light source, with typical fwhm bandwidths ca. 16 nm using a 1200 g mm^–1^, 750 nm blaze grating. A single monochromator was used for detection (Princeton HRS-300) with 1200 g mm^–1^ (500 nm blaze) and 150 g mm–1 (800 nm blaze) gratings used for measuring vis-NIR and SWIR spectra, respectively. Typical exposures were 0.5–1 s with 0.25–1 mm detection slit widths. PL spectra for each solution were excited at 500 and 770 nm with identical spectral results. Experiments were run in 2 mm cuvettes utilizing front-face geometry to maximize signal and minimize scatter.

A Coherent Libra Ti:sapphire laser with a repetition rate of 1 kHz and a fundamental wavelength of 800 nm (100 fs pulse width) was used for ultrafast transient absorption experiments. The probe pulse (λ probe = 400 to 1650 nm) was generated by focusing a small portion of the Libra output into a sapphire crystal. The probe pulse was focused at the sample and pump, probe pulses were spatially overlapped, and a mechanical delay stage was used to delay the probe pulse relative to the pump. The time window for the experiment is 5 ns. A small portion of the probe was redirected before the sample to be used as a reference to reduce noise. For the ns-μs experiments, an electronically delayed supercontinuum probe with roughly 1 ns time resolution was used to generate the probe pulse instead. Changes in the probe spectrum were monitored through a fiber optic coupled multichanneled spectrometer with a CMOS sensor. Helios, EOS, and Surface Xplorer software from Ultrafast Systems were used to collect and process the data.

## Supplementary Material



## Notes

TEM imaging, further analysis of UV–vis Absorbance, ^1^NMR, TA fluence measurements, TA kinetics, further TA spectra, non-normalized PL data, FTIR data on films and comparing different systems, TA global fit curves, theory results for PbS/OA, initial geometries for PbS/Tc-DA systems, and charge density difference calculations (PDF)
