# Supplementary material for: Interligand Coupling Drives Fast Triplet Energy Transfer Routes in PbS/Tetracene Quantum Dot Hybrids
Source: ACS Nano. 2025 Nov 14;19(46):40245–57. doi: 10.1021/acsnano.5c16982 (PMC12659426; doi:10.1021/acsnano.5c16982)
Supplement: Supplementary file 1 [file nn5c16982_si_001.pdf]

Supporting Information for:

Inter-ligand coupling drives fast triplet energy transfer routes in PbS/tetracene quantum dot hybrids

Benjamin Feingold,[1,2] Nicholas F. Pompetti,[1] Marissa Martinez,[1] Taylor J. Aubry,[1] Jeffrey L. Blackburn,[1] Obadiah G. Reid,[1] Matthew C. Beard,[1,*] Justin C. Johnson[1,*]

[1]Chemistry and Nanoscience Center, National Renewable Energy Laboratory, 15013 Denver West Pkwy, Golden, CO 80401, United States

[2]Department of Chemistry, University of Colorado, Boulder 80305, United States

*Address correspondence to Matt.Beard@nrel.gov Justin.Johnson@nrel.gov

Contents

TEM Images: 2-5

UV-Vis Absorbance: 6-10

[1]NMR: 11-13

FTIR: 14-19

Theory: 20-21

TA Spectra: 22-24, 26-29

PL Spectra: 25

TA Global Fits: 30-32

TEM

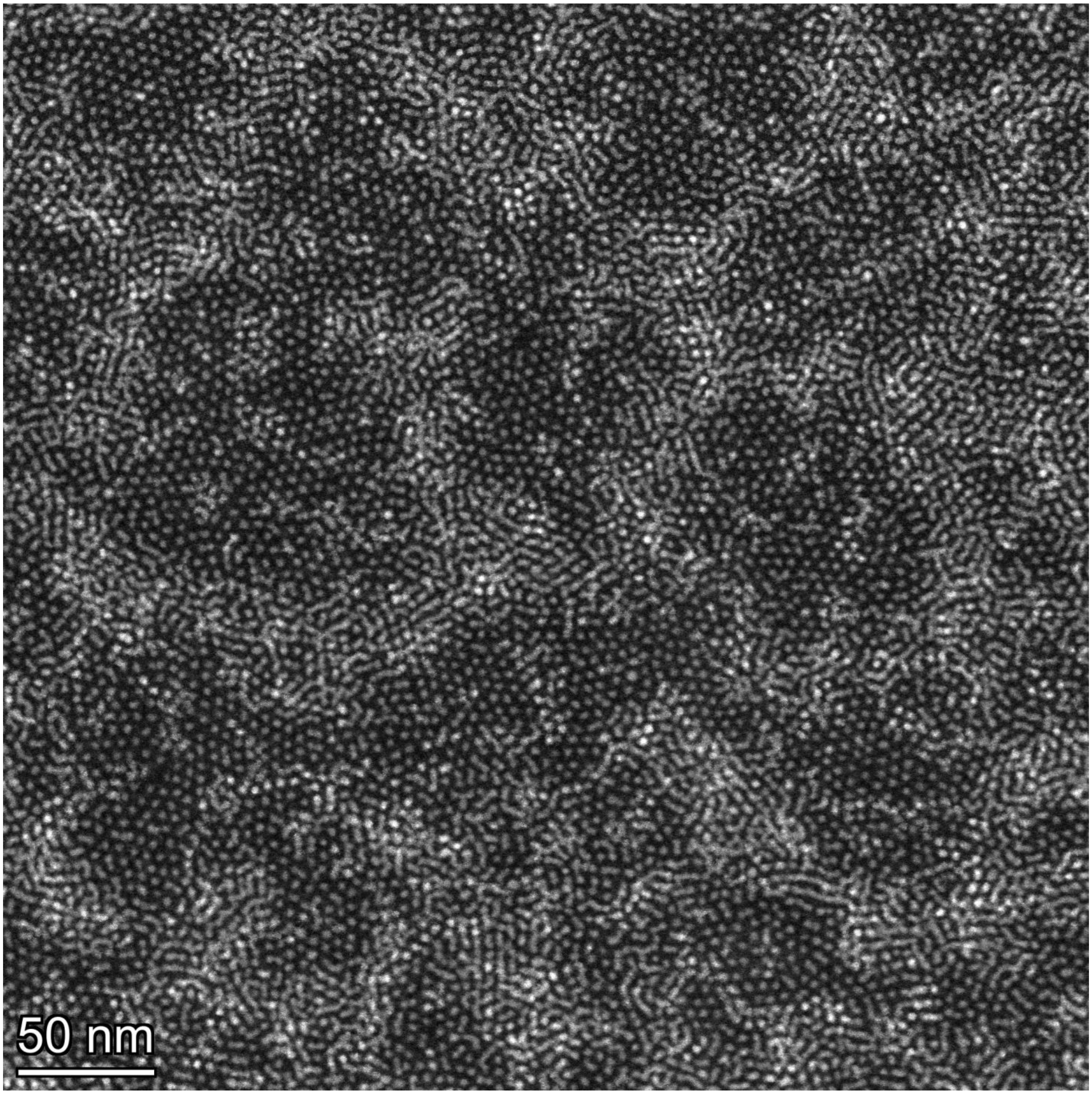

Figure S1: TEM image of PbS/Oleate on Formvar / Carbon 200 Mesh grid. The TEM grid was densely coated with more than a single layer of QD deposited in many areas. TEM grids were plasma cleaned to remove carbon contamination, which may result in QD fusing as passivating oleate ligands are removed.

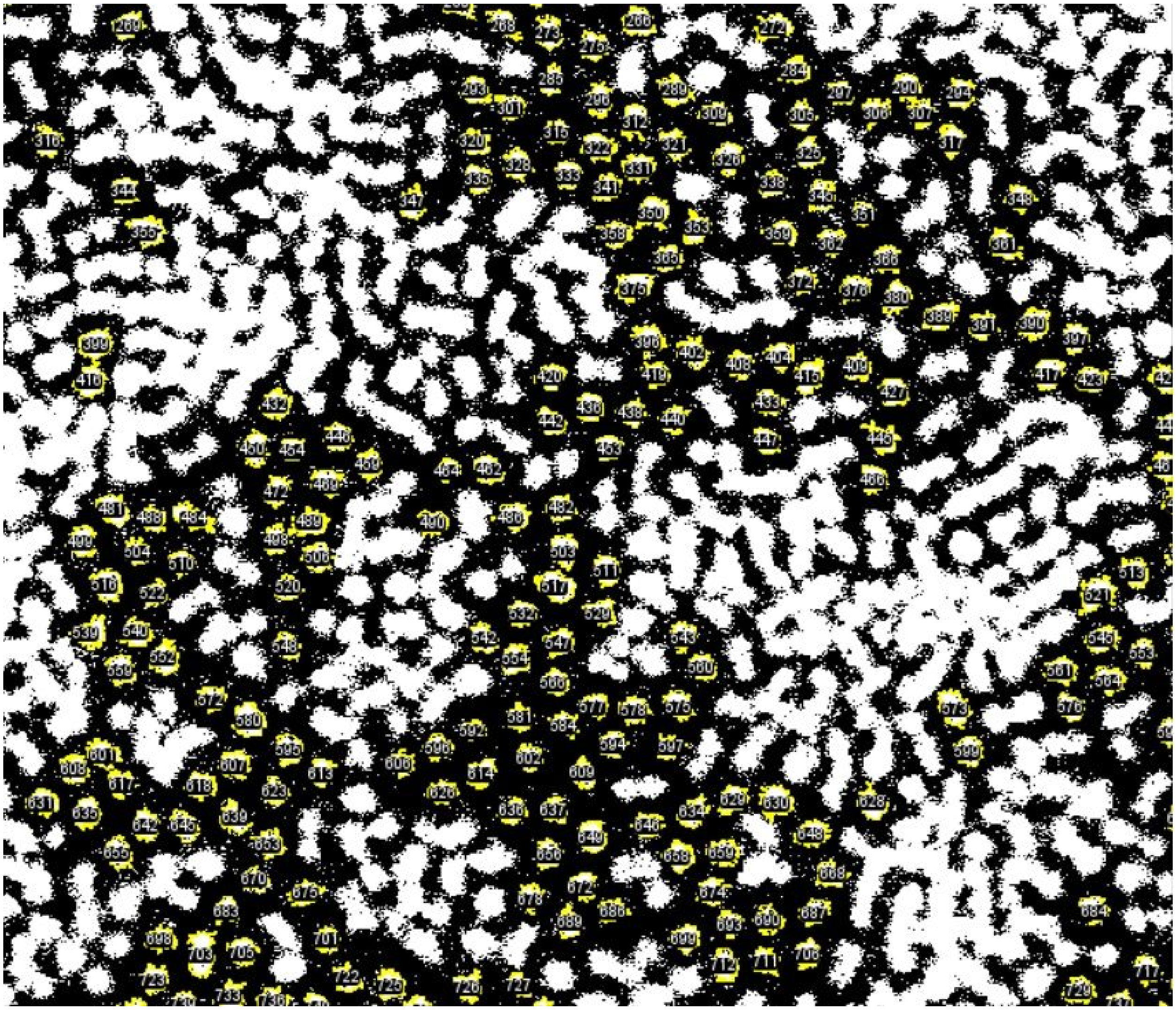

Figure S2: Zoomed in view of particle analysis performed via ImageJ (FIJI). Large clusters and areas where QDs fused were excluded to avoid bias towards a larger QD diameter.

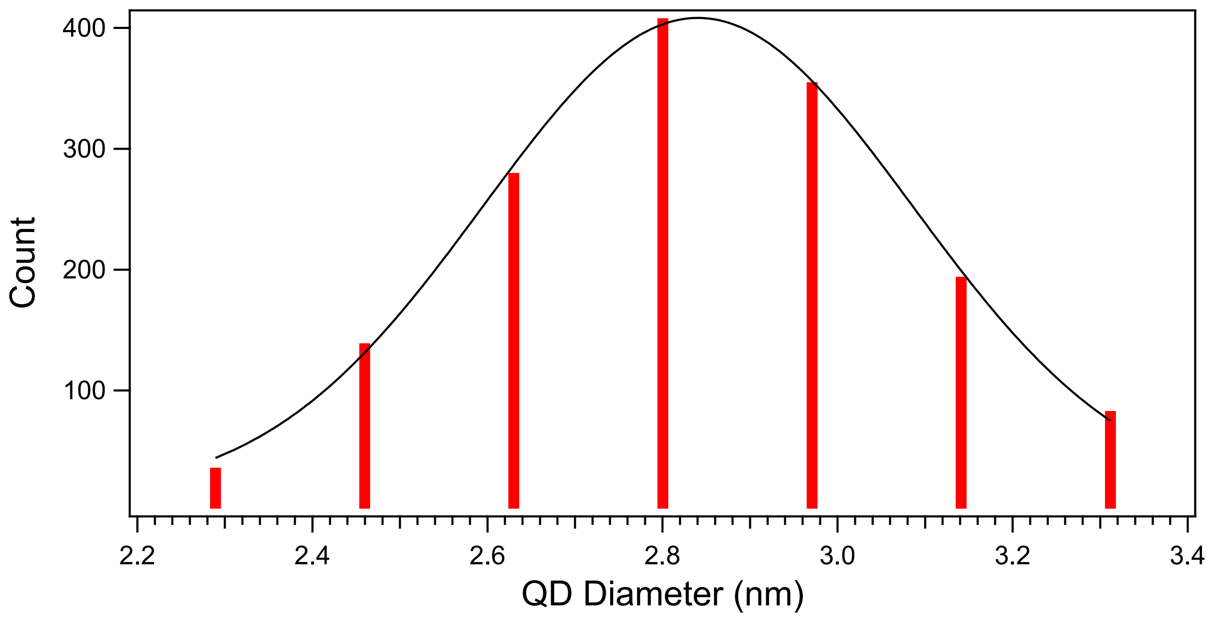

Figure S3: Results of particle analysis fit to a gaussian curve finds an average QD diameter of 2.85 ± 0.28 nm. The polydispersity found here is larger than expected from the width of the exciton in absorbance spectroscopy. This is likely a result of the very small size of the QDs leading to lower TEM resolution, the density of QDs deposited on the TEM grid, and the fusing of QDs both from being cast into films and from plasma cleaning.

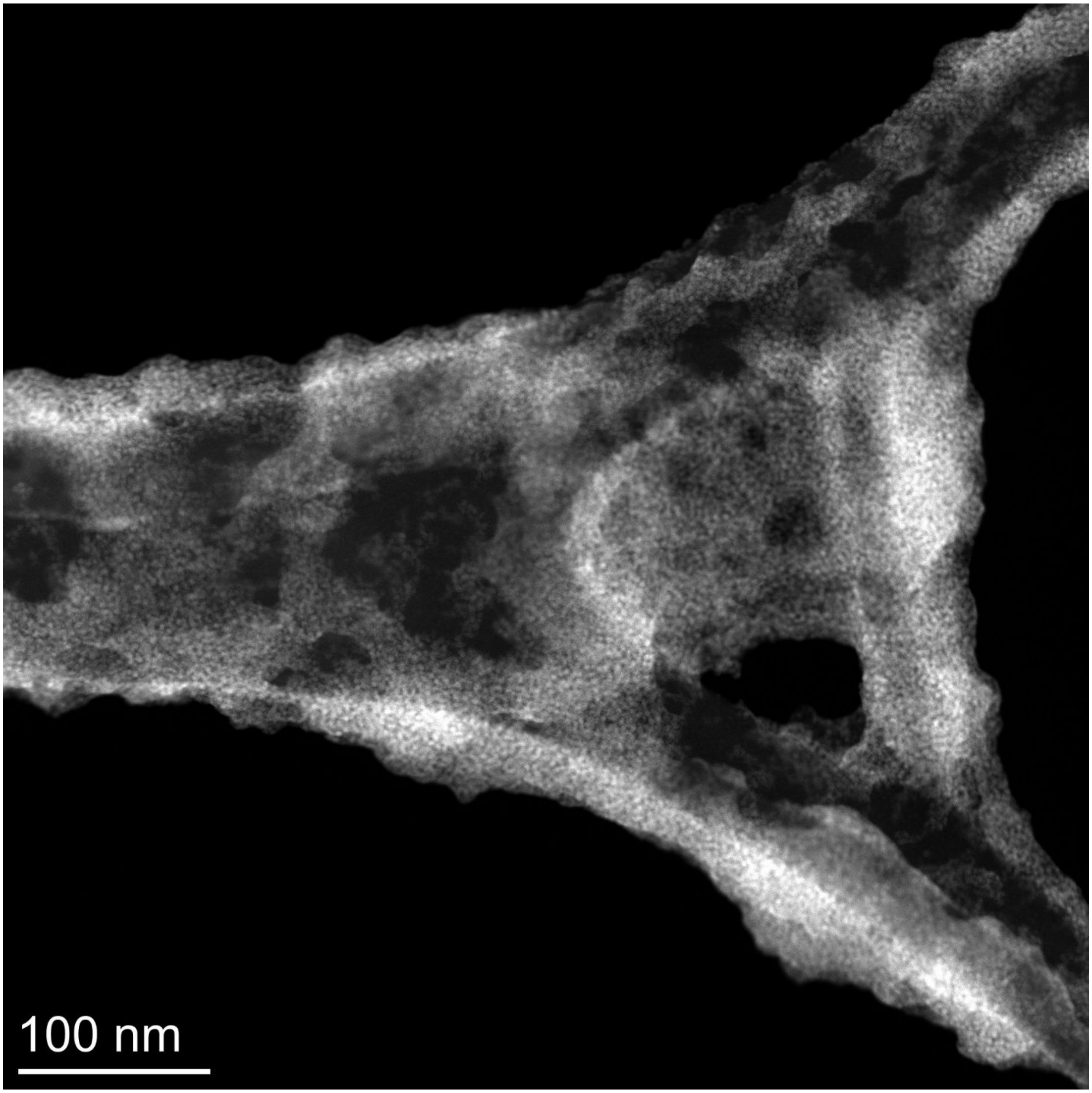

Figure S4: TEM image of 1 mM PbS/Tc-DA on Lacey Formvar / Carbon 200 Mesh grid. QDs densely cluster into regions rather than forming layers like oleate-capped QDs. This suggests an increased affinity of PbS/Tc-DA for each other. The intense clustering prevents particle size analysis.

Absorbance

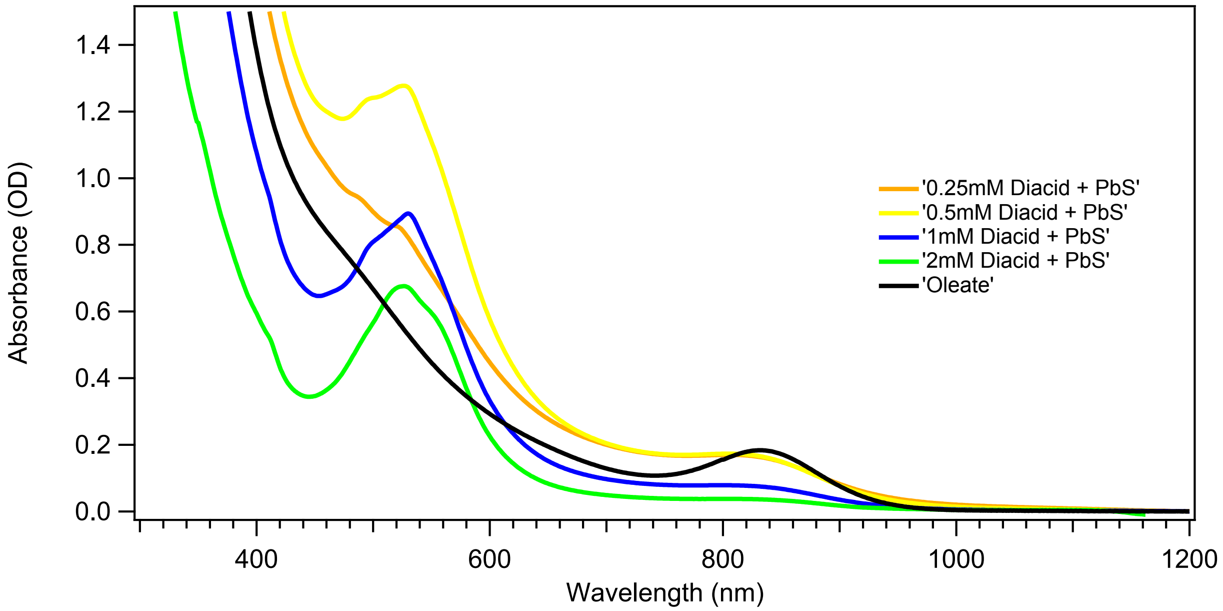

Figure S5: Absorption spectra of PbS/Tc-DA exchanged with varying concentrations of Tc-DA. PbS/Oleate spectrum included for reference. 0.25, 0.5, and 1 mM were collected in a 2 mm quartz cuvette. 2 mM was collected in a 1 mm quartz cuvette. 1 mM and 2 mM samples were diluted by half to avoid saturation of the spectrometer.

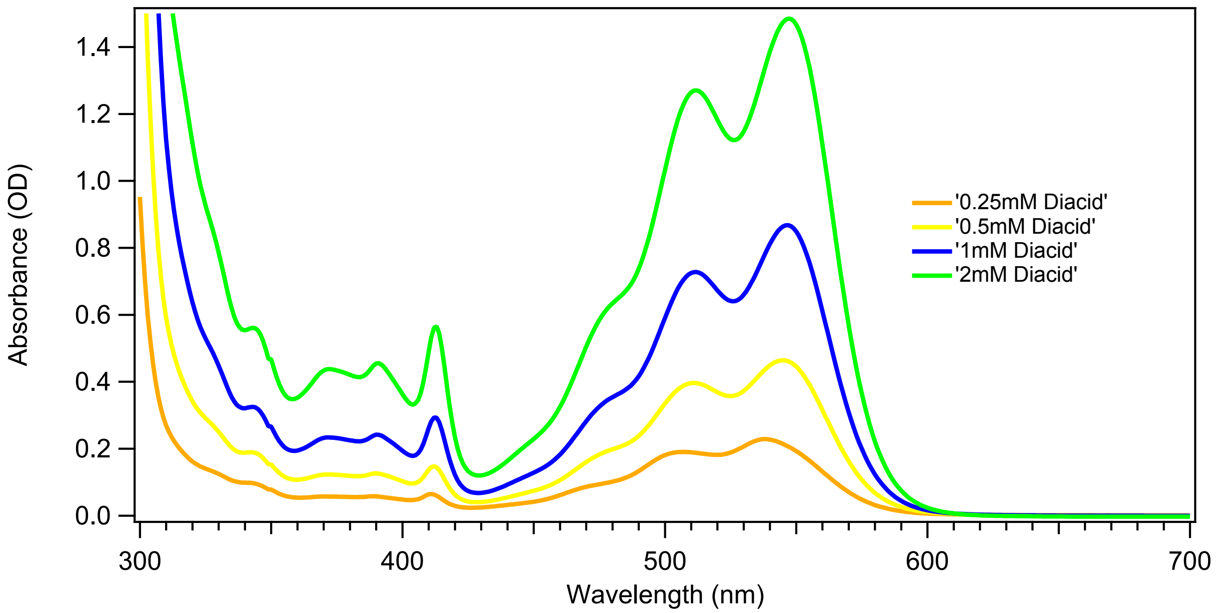

Figure S6: Absorption spectra of neat Tc-DA in DMF at varying concentrations. Spectra were collected in a 1 mm quartz cuvette. Tc-DA exhibits a complex aggregation scheme with respect to concentration which results in a highly non-linear extinction coefficient at different concentrations.[1]

830nm PbS Concentration (uM)		2mM Diacid (M)	1mM Diacid (M)	0.5mM Diacid (M)	0.25mM Diacid (M)
615.7927		0.002	0.001	0.0005	0.00025
Amount Added (uL)		Volume (uL)	Volume (uL)	Volume (uL)	Volume (uL)
25		500	500	500	500
Diluted To (uL)		Amount of Diacid (mc	Amount of Diacid (mol	Amount of Diacid (mol)	Amount of Diacid (mol)
500		0.000001	0.0000005	0.00000025	0.000000125
Diluted Concentration (uM)		Amount of Diacid (pa	Amount of Diacid (part	Amount of Diacid (partic	Amount of Diacid (particles)
30.78963		6.02E+17	3.011E+17	1.5055E+17	7.52768E+16
Diluted Concentration (M)		Ratio Diacid:QD	Ratio Diacid:QD	Ratio Diacid:QD	Ratio Diacid:QD
3.08E-05		64.957	32.478	16.239	8.120
Amount of QD (mol)					
1.54E-08					
Amount of QD (particles)					
9.27E+15					

Table S1: Calculated ratio of molecules Tc-DA to QDs for 2 (green), 1 (blue), 0.5 (yellow), and 0.25 mM (orange) PbS/Tc-DA solution. This represents an upper bound on the possible quantity of Tc-DA bound to the QD surface. It is highly unlikely that every possible Tc-DA molecule in solution is bound to a QD.

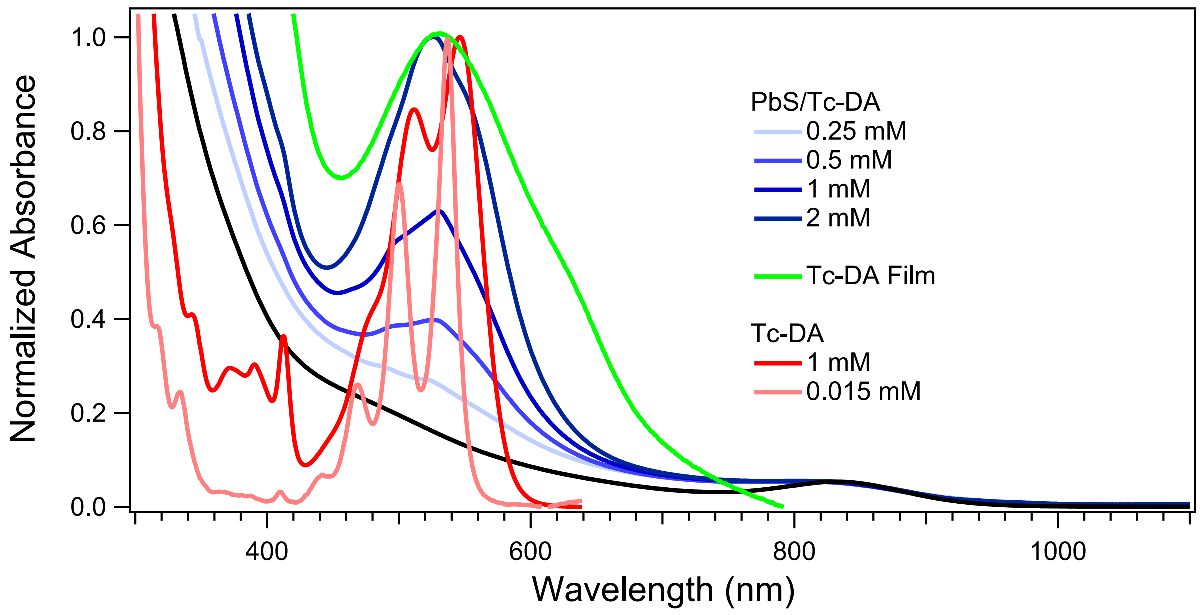

Figure S7: Normalized absorption spectra of PbS/Oleate (black), 0.015 and 1mM Tc-DA in DMF (red), a film of neat Tc-DA (green), and PbS/Tc-DA ligand exchanged with varying concentrations of Tc-DA (blue).

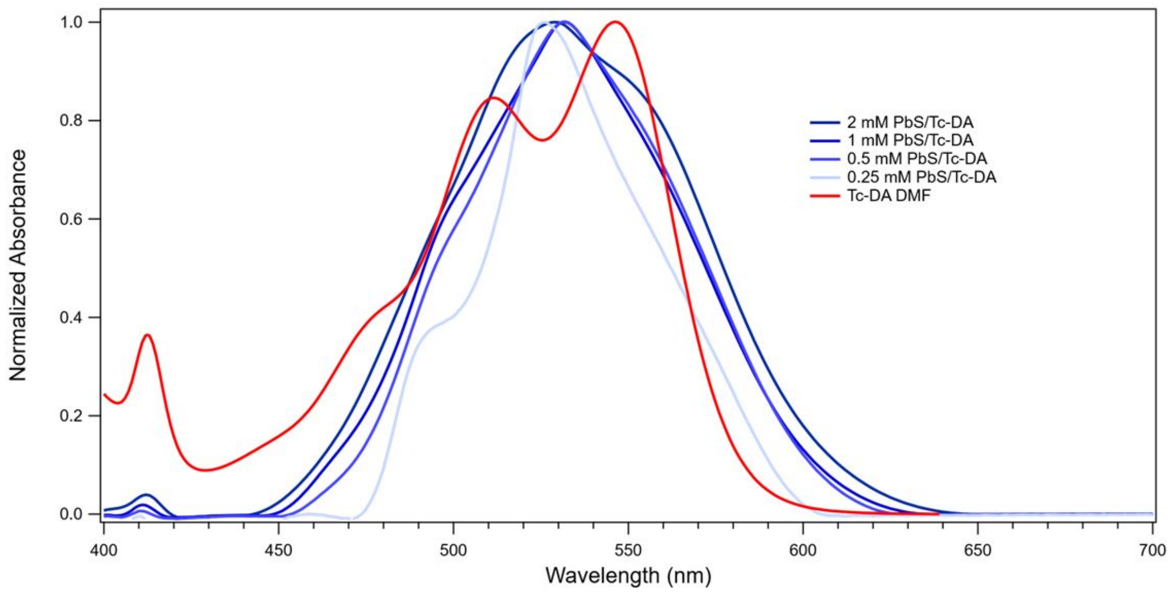

Figure S8: Absorption spectra of PbS/Tc-DA exchanged with varying concentrations of Tc-DA while subtracting out the QD tail absorbance (blue). 1 mM Tc-DA in DMF is shown in red for comparison.

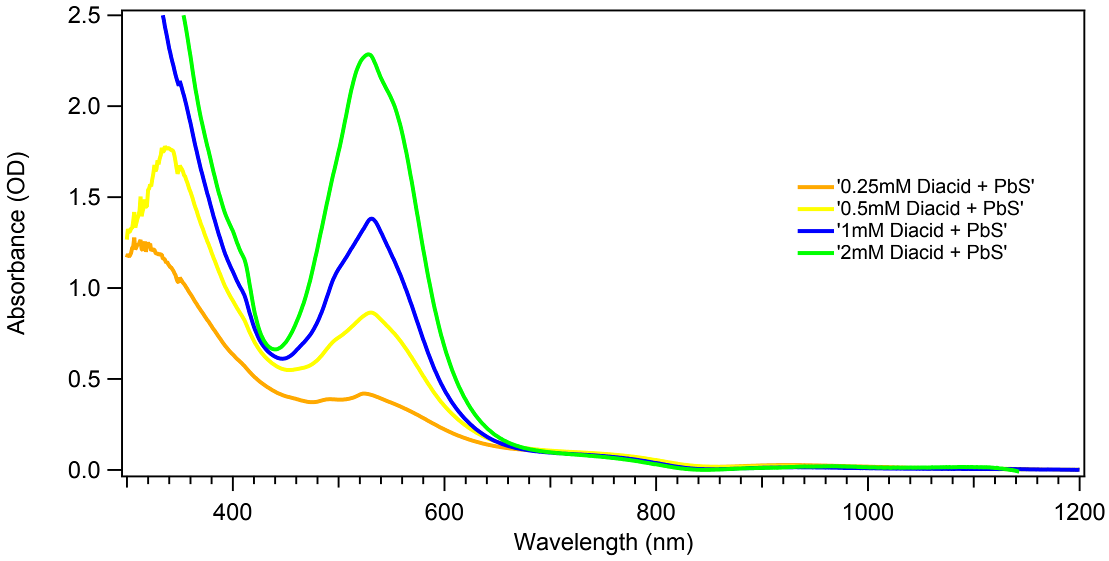

Figure S9: Absorption spectra of PbS/Tc-DA exchanged with varying concentrations of Tc-DA. 2 mM was multiplied by 4 and 1 mM by 2 compared to S5 to account for dilution and cuvette pathlength. Then the PbS/Oleate spectrum was subtracted from the Tc-DA exchanged spectra. This should result in spectra that represent the Tc-DA on the QD surface and the broad-band absorbance enhancement.

	2mM	1mM	0.5mM	0.25mM		
0-0 (OD)	1.4844	0.86728	0.46399	0.22849		
0-1 (OD)	1.2703	0.7274	0.39621	0.19014		
0-2 (OD)	0.60761	0.33231	0.17864	0.089318		
	Molarity	Molarity	Molarity	Molarity		
	0.002	0.001	0.0005	0.00025		
0-0 Extinction M−1·cm−1	7422	8672.8	9279.8	9139.6		
0-1 Extinction M−1·cm−1	6351.5	7274	7924.2	7605.6		
0-2 Extinction M−1·cm−1	3038.05	3323.1	3572.8	3572.72		
	With QD					
Ligand Absorbance Maximum (OD)	2.2845	1.3807	0.86473	0.41772		
Concentration Diacid	0.003078	0.00159199	0.000932	0.000457044	Amount of QD (particles)	
Amount of Diacid (mol)	1.54E-06	7.9599E-07	4.66E-07	2.28522E-07	9.27E+15	
Amount of Diacid (particles)	9.27E+17	4.7936E+17	2.81E+17	1.37619E+17		
Ratio Diacid:QD	99.969	51.705	30.265	14.844		

Table S2: Calculated extinction coefficients for Tc-DA in DMF at 2 (green), 1 (blue), 0.5 (yellow), and 0.25 mM (orange). As noted in Figure S6, the extinction coefficient changes with concentration. The 0-1 extinction coefficient was used to calculate the amount of Tc-DA molecules bound to the QD surface based on the maximum absorbance of Tc-DA in Figure S7 (~530 nm). The calculated amount of Tc-DA on the QD surface exceeds the theoretical maximum shown in Table S1. This again highlights the strong absorbance enhancement and distortion of the Tc-DA spectra caused by binding to the PbS QD.

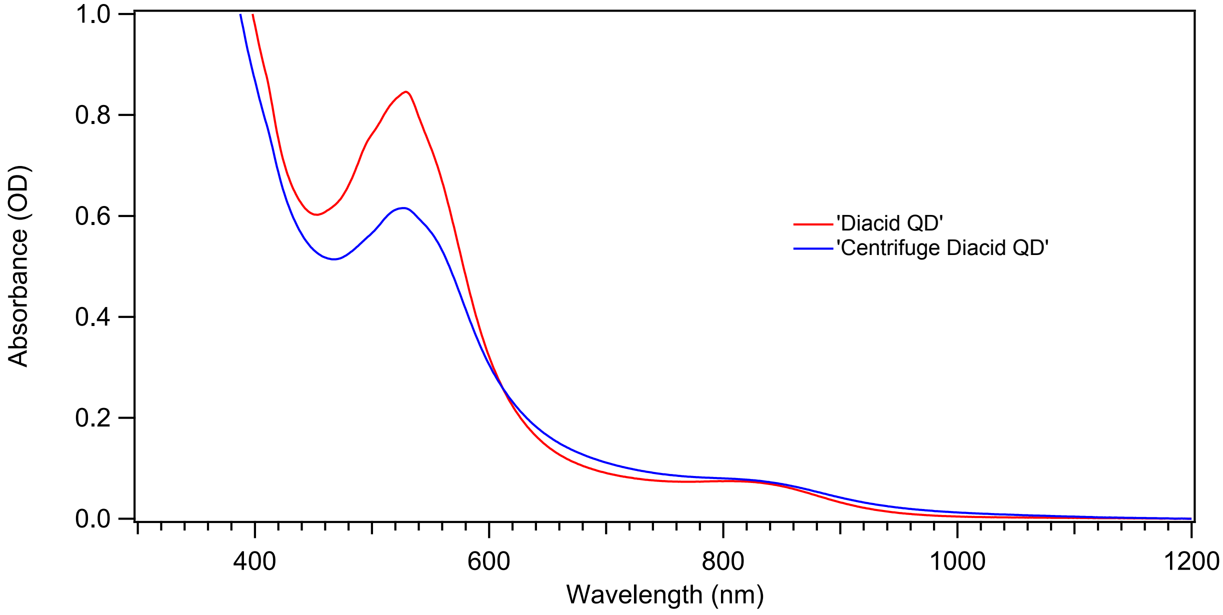

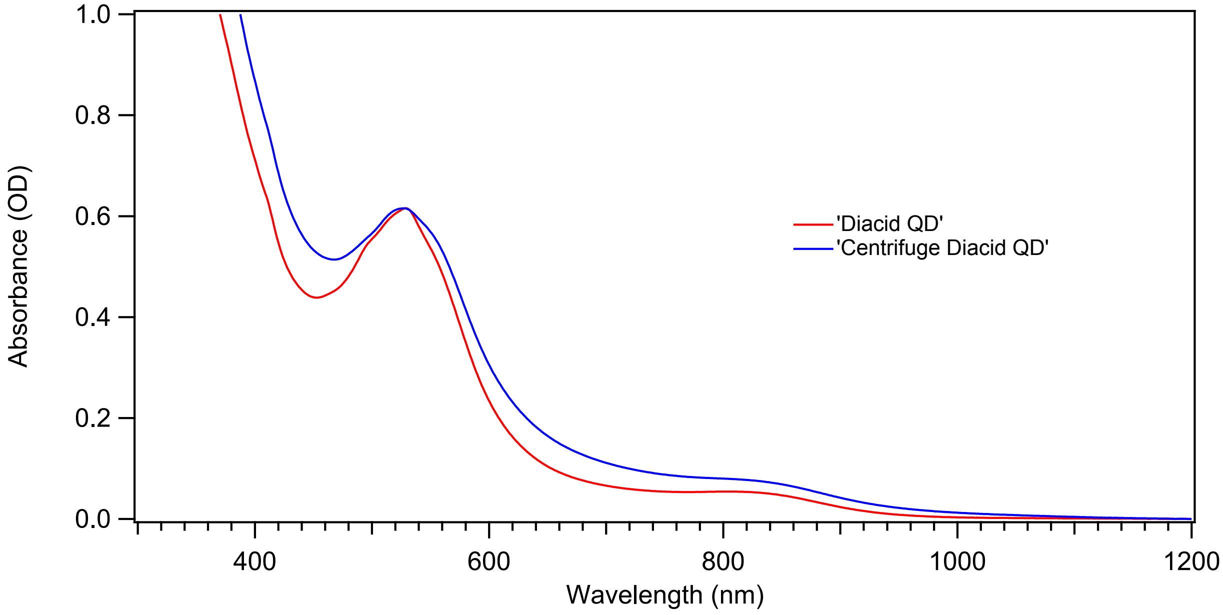

Figure S10: Absorption spectra for 1 mM PbS/Tc-DA before and after centrifugation. The top panel shows normalization to the QD exciton (~830 nm) while the bottom shows normalization to the Tc-DA band (~530 nm). There is a loss of absorbance intensity post-centrifugation which is likely attributable to dilution and loss of material upon resuspension. The Tc-DA band and QD exciton maintain very similar structures pre and post-exchange, suggesting that centrifugation does not result in substantial removal of any species present in the solution.

^{1}NMR

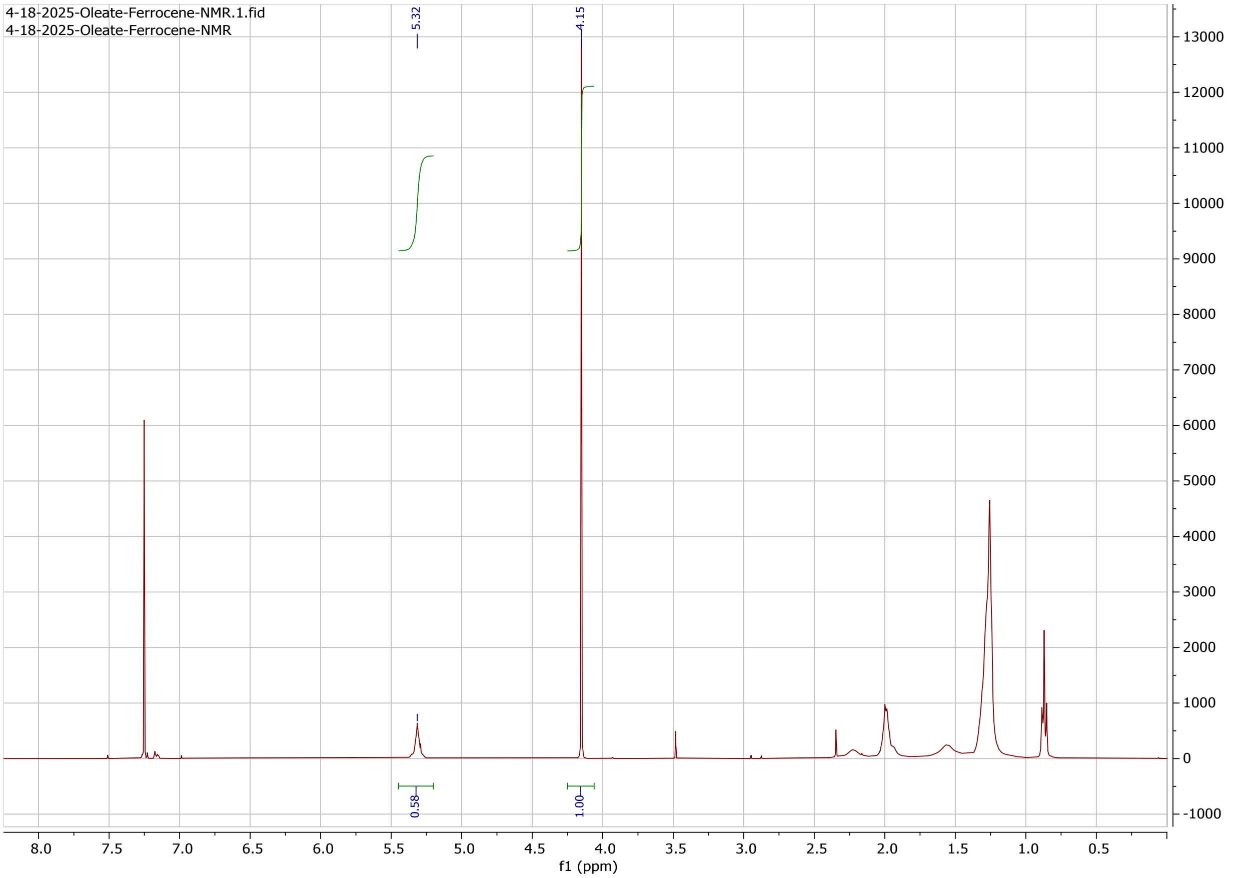

Figure S11: 400 MHz ^{1}HNMR Spectrum of PbS/Oleate in $CDCl_3$. A known quantity of ferrocene was included as an analytical standard (4.15 ppm peak). The peak at 5.3 ppm represents the protons of the oleate alkene which is used to calculate the amount of oleates bound to each QD. The peak at 7.26 ppm is trace $CHCl_3$. The collection of broad peaks from 0.6-3.5 ppm are various protons on the oleate alkanes. Being bound to a QD results in slow tumbling of the oleate protons during the NMR experiment which leads to weaker, broader signals.

QD Stock Concentration (uM)	Molarity of Stock Ferrocene (M)	QD Vinyl Peak Integration	Oleate per QD	QD Diameter (nm)
615.79	0.05	0.58	94.18795	2.68
Volume QD in NMR Tube (uL)	Volume Ferrocene in NMR Tube (uL)	Ferrocene Peak Integration		Radius (nm)
50	20	1		1.34
Total Volume in NMR Tube (uL)	Ferrocene Conentration in NMR tube (M)	Oleate Concentration (M)		Surface Area (nm^2)
500	0.002	0.0058		22.56418
QD Concentration in NMR Tube (uM)	Ferrocene Conentration in NMR tube (mM)	Oleate Concentration (um)		Oleate per nm^2
61.579	2	5800		4.174225
QD Concentration in NMR Tube (mM)				
0.061579				

Table S3: Calculation of amount of oleates per QD based on known values of QD and ferrocene concentration for PbS/Oleate. Ferrocene has 10 identical protons while the oleate alkene has 2 identical protons.

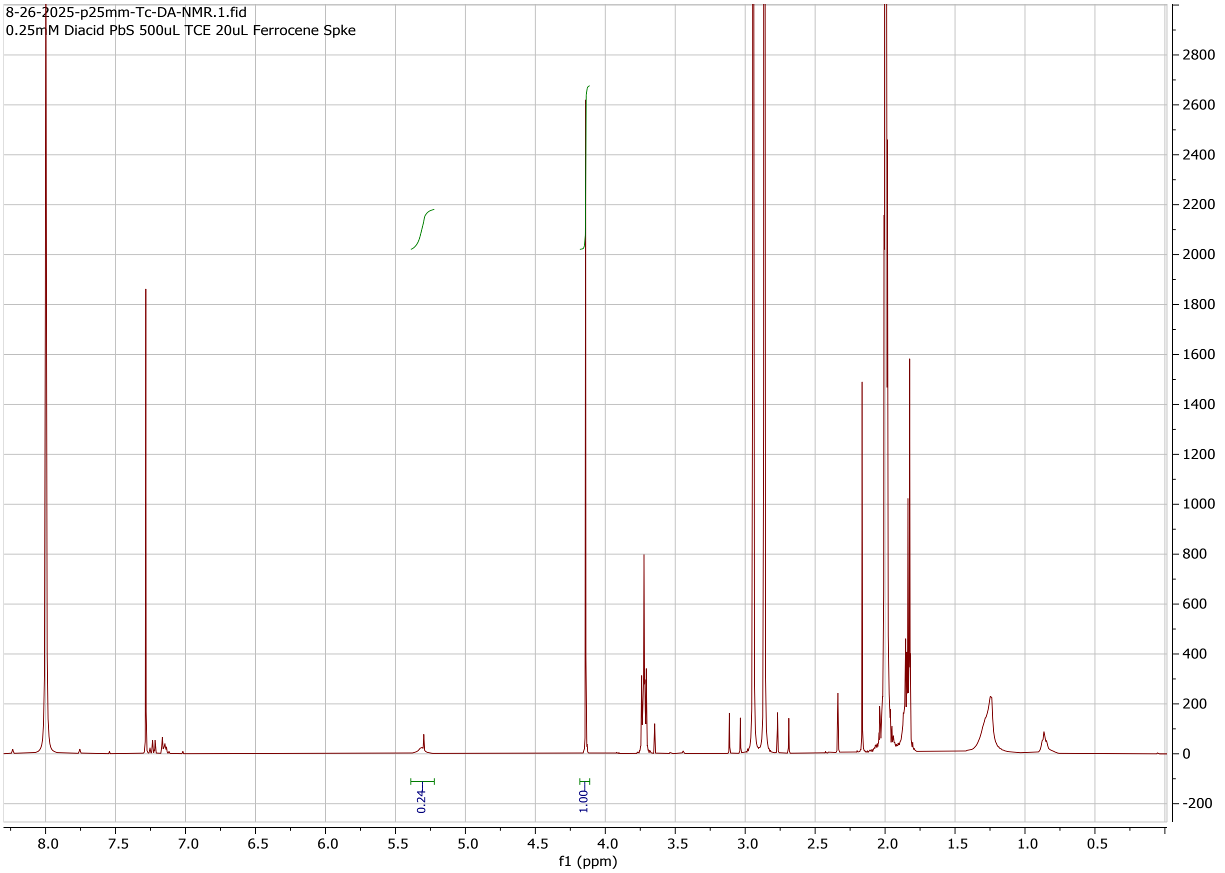

Figure S12: 400 MHz ^{1}HNMR Spectrum of 0.25 mM PbS/Tc-DA in $CDCl_3$. The peaks at 8, 2.95, and 2.88 ppm represent trace amounts of DMF remaining after centrifugation and resuspension in $CDCl_3$. The peak at 5.3 ppm is attenuated compared to unexchanged samples, signaling the replacement of oleate by Tc-DA. There are many new peaks from 0.6-3.5 ppm when compared to the oleate sample, but the shifting and broadening that occurs when binding the QD surface make it virtually impossible to identify these protons. See Pompetti JACS 2024 for NMR of neat Tc-DA.[1]

QD Stock Concentration (uM)	Molarity of Stock Ferrocene (M)	QD Vinyl Peak Integration	Oleate per QD	QD Diameter (nm)
615.79	0.05	0.24	77.94865	2.68
Volume QD in NMR Tube (uL)	Volume Ferrocene in NMR Tube (uL)	Ferrocene Peak Integration		Radius (nm)
25	20	1		1.34
Total Volume in NMR Tube (uL)	Ferrocene Contentration in NMR tube (M)	Oleate Concentration (M)		Surface Area (nm^2)
500	0.002	0.0024		22.56418
QD Concentration in NMR Tube (uM)	Ferrocene Contentration in NMR tube (mM)	Oleate Concentration (um)		Oleate per nm^2
30.7895	2	2400		3.454531
QD Concentration in NMR Tube (mM)				
0.03079				

Table S4: Calculation of amount of oleates per QD based on known values of QD and ferrocene concentration for 0.25 mM PbS/Tc-DA. There is an ~17% reduction in the number of oleates per QD post exchange.

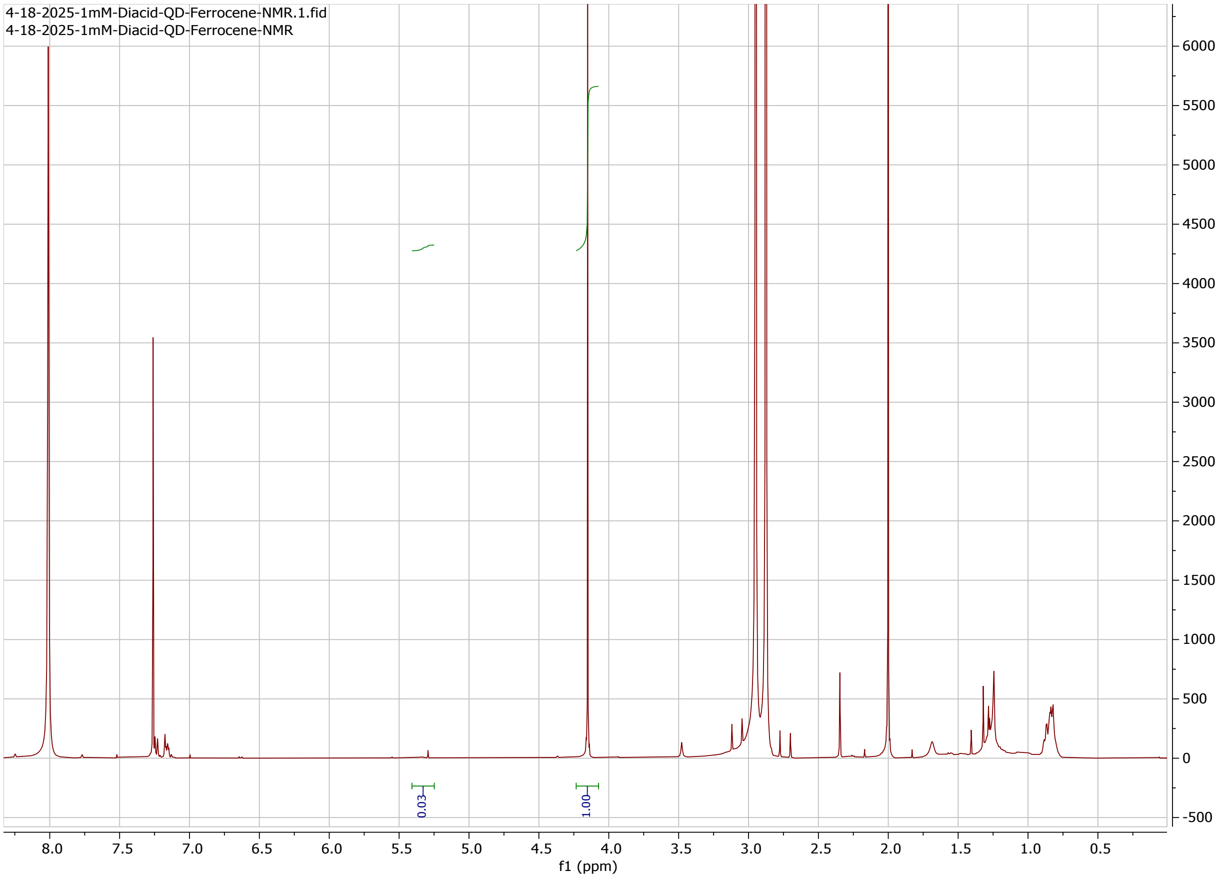

Figure S13: 400 MHz ^{1}HNMR Spectrum of 1 mM PbS/Tc-DA in $CDCl_3$. The peaks at 8, 2.95, and 2.88 ppm represent trace amounts of DMF remaining after centrifugation and resuspension in $CDCl_3$. The peak at 5.3 ppm is substantially attenuated compared to unexchanged samples, signaling the replacement of oleate by Tc-DA. There are many new peaks from 0.6-3.5 ppm when compared to the oleate sample, but the shifting and broadening that occurs when binding the QD surface make it virtually impossible to identify these protons. See Pompetti JACS 2024 for NMR of neat Tc-DA.[1]

QD Stock Concentration (uM)	Molarity of Stock Ferrocene (M)	QD Vinyl Peak Integration	Oleate per QD	QD Diameter (nm)
615.79	0.05	0.03	12.99144	2.68
Volume QD in NMR Tube (uL)	Volume Ferrocene in NMR Tube (uL)	Ferrocene Peak Integration		Radius (nm)
18.75	20	1		1.34
Total Volume in NMR Tube (uL)	Ferrocene Conentration in NMR tube (M)	Oleate Concentration (M)		Surface Area (nm^2)
500	0.002	0.0003		22.56418
QD Concentration in NMR Tube (uM)	Ferrocene Conentration in NMR tube (mM)	Oleate Concentration (um)		Oleate per nm^2
23.09213	2	300		0.575755
QD Concentration in NMR Tube (mM)				
0.023092				

Table S5: Calculation of amount of oleates per QD based on known values of QD and ferrocene concentration for 1 mM PbS/Tc-DA. There is an ~86% reduction in the number of oleates per QD post exchange.

FTIR

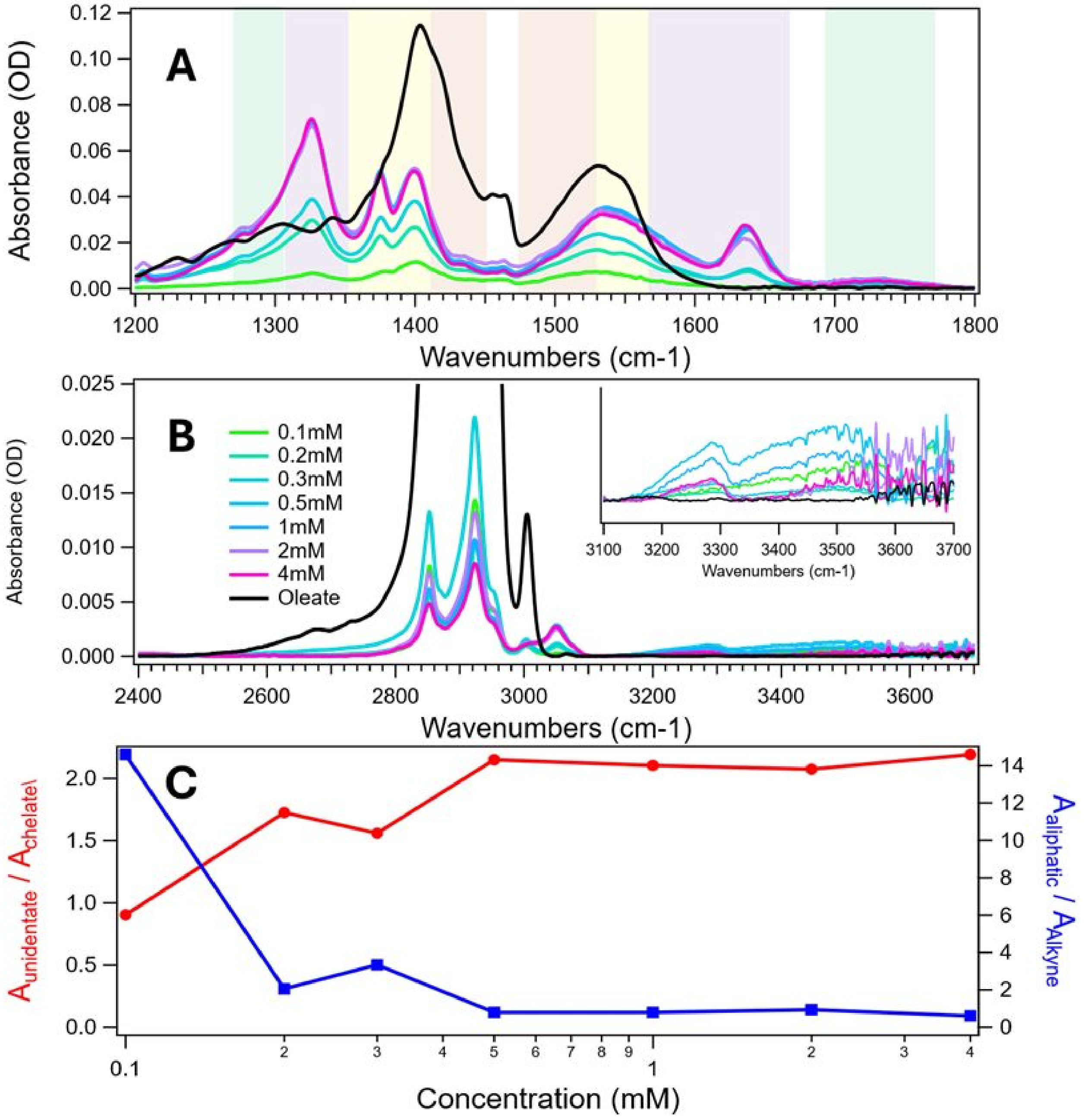

Figure S14: FTIR spectra of the (A) carboxylate and (B) aliphatic regions for films of PbS/Oleate and for PbS/Tc-DA at varying concentrations of Tc-DA. Colored highlighting indicates the features arising from chelating (orange), bridging (yellow), unidentate (purple), and free COOH (green) motifs. (C) Ratio between the integrated areas for unidentate and chelate + bridging peaks (red circles) and for aliphatic and alkyne peaks (blue squares).

The carboxylate region (Figure S25A) encompasses the symmetric modes from 1200-1450 cm^{-1} and the asymmetric modes from 1450-1700 cm^{-1}.[2,3] The PbS/Oleate spectrum is dominated by a

chelating and bridging mode with symmetric peaks at 1419 and 1403 cm^{-1} and asymmetric peaks at 1549 and 1529 cm^{-1}. These peaks are similar to those previously found for PbS/Oleate, but are shifted by a few wavenumbers, likely as a result of the smaller size of QD used here.[2,4] Upon exchange with Tc-DA, the chelating mode is heavily suppressed while another bridging mode (1558 and 1374 cm^{-1}) and two unidentate modes (1590 and 1328 cm^{-1}, 1635 and 1319 cm^{-1}) grow in. Relatively weak free COOH modes also emerge after the exchange (1732 and 1288 cm^{-1}). All features are in line with previous results from exchanging Tc-Diacid onto larger QDs,[2] although trends with concentration are altered. For films of PbS/Tc-DA, there appears to be increasing exchange of oleate for Tc-DA up to 0.5 mM. At concentrations 0.5 mM and higher, we observe no substantial differences in intensity for the Tc-DA associated bridging peak or for the free COOH peaks. Our previous studies with Tc-DA on larger PbS had shown that trends in the relative strengths of these features as a function of concentration implicated a distribution of ligand geometries (face-on v. edge-on) that trended toward face-on at highest ligand loadings.[2] Based on the consistent relative intensities of peaks here, we conclude that there is no clear analogy for small PbS QDs as a function of Tc-DA exchange concentration in films.

The aliphatic region (Figure S25B) is dominated by C-H stretches associated with residual oleates from 2750-3000 cm^{-1}.[4] Consistent with results from exchange on large QDs, we observe a nearly order of magnitude reduction in oleate signals upon exchange with even the lowest concentrations of Tc-DA.[2] The oleate signals are further reduced by exchanges with higher concentrations of Tc-DA. After exchange, we also observe very weak signals associated with OH stretches from 3200-3600 cm^{-1}. These signals are relatively narrow compared to what would be expected if there was significant hydrogen bonding and are a good match for the previously observed parallel Tc-DA geometry, allowing a tentative assignment of similar face-on geometries here, particularly at low concentrations.[2,5–7]

Figure S25C shows the ratios between the integrated areas of the unidentate and chelate peaks (red circles) and the aliphatic and alkyne peaks (blue squares) for films, which equally demonstrate extent of exchange and its effective saturation at 0.5 mM and higher.

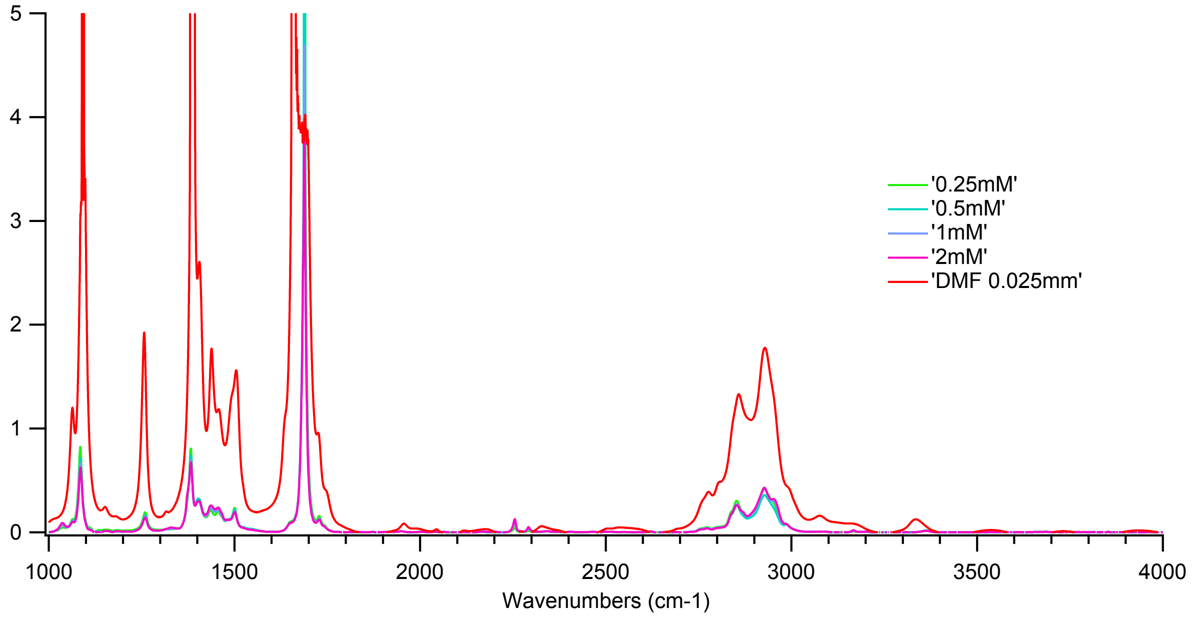

Figure S15: FTIR spectrum showing absorbance of DMF compared to that of the PbS/Tc-DA exchanged with varying concentration of Tc-DA. QD experiments were performed using a 0.5 mm pathelength. The red trace shows DMF with a 0.025 mm pathlength. DMF features strongly compete with those of the exchanged QDs and thus it is very difficult to distinguish the origin of the peaks.

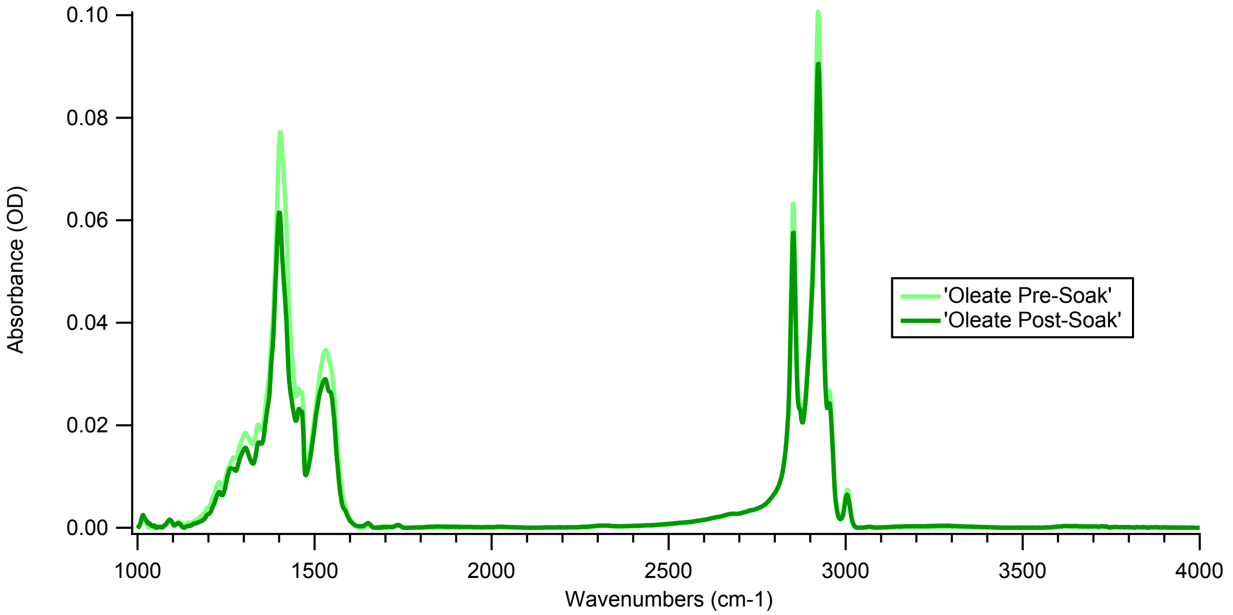

Figure S16: FTIR spectra of PbS/Oleate film before and after soaking for 1 hour in neat DMF. The loss of signal is much less than for exchange with Tc-DA and there are no new peaks or shifts of existing peaks.

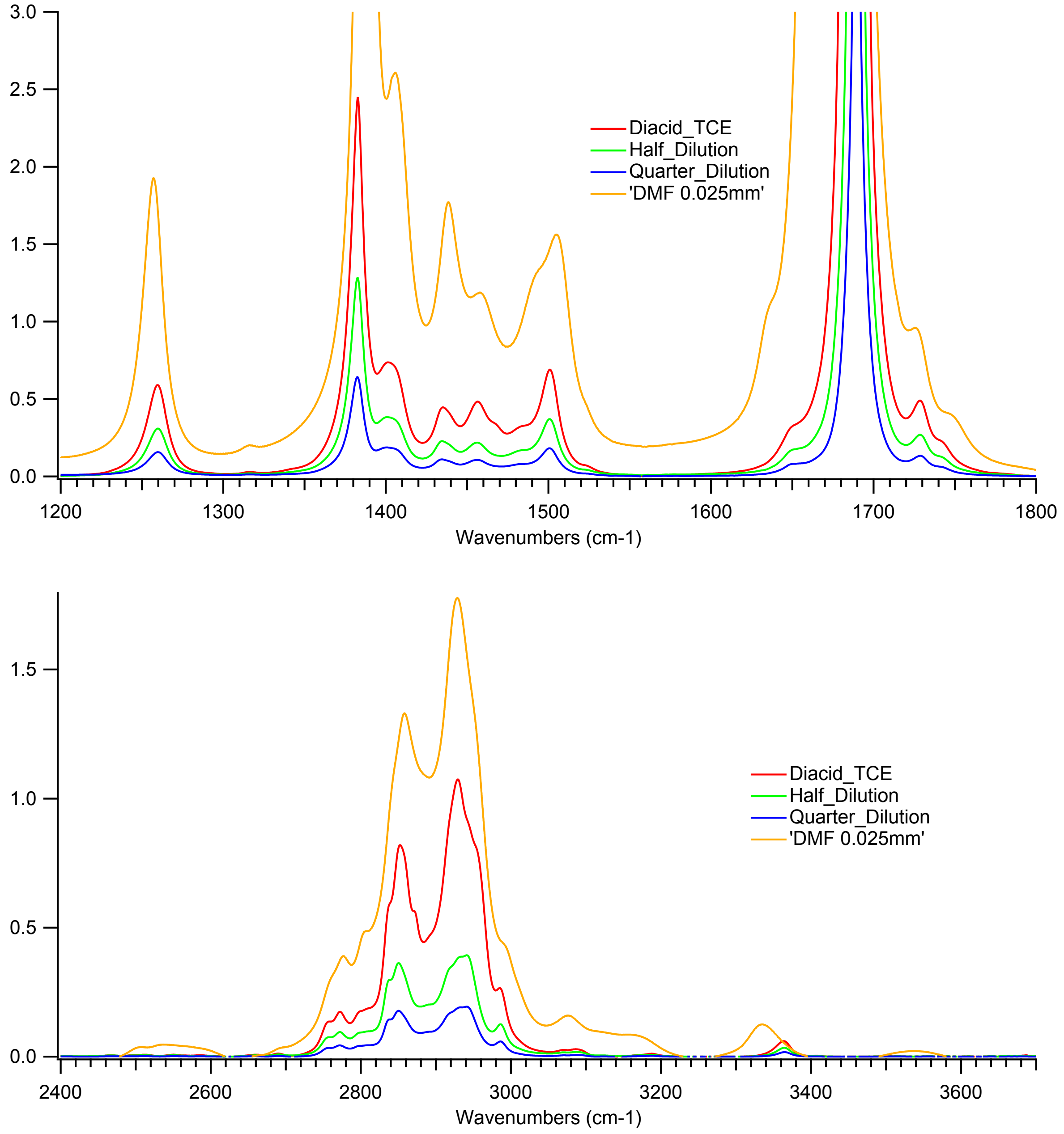

Figure S17: FTIR spectra of carboxylate (top) and aliphatic (bottom) regions for solutions of neat Tc-DA in TCE with ~1.3% DMF for solubility. Neat Tc-DA has no aliphatic groups, and thus all of the signal in that region must come from DMF. The excellent overlap between the DMF peaks and the neat Tc-DA peaks in the carboxylate region also suggests that most of the ‘signal’ is coming from DMF.

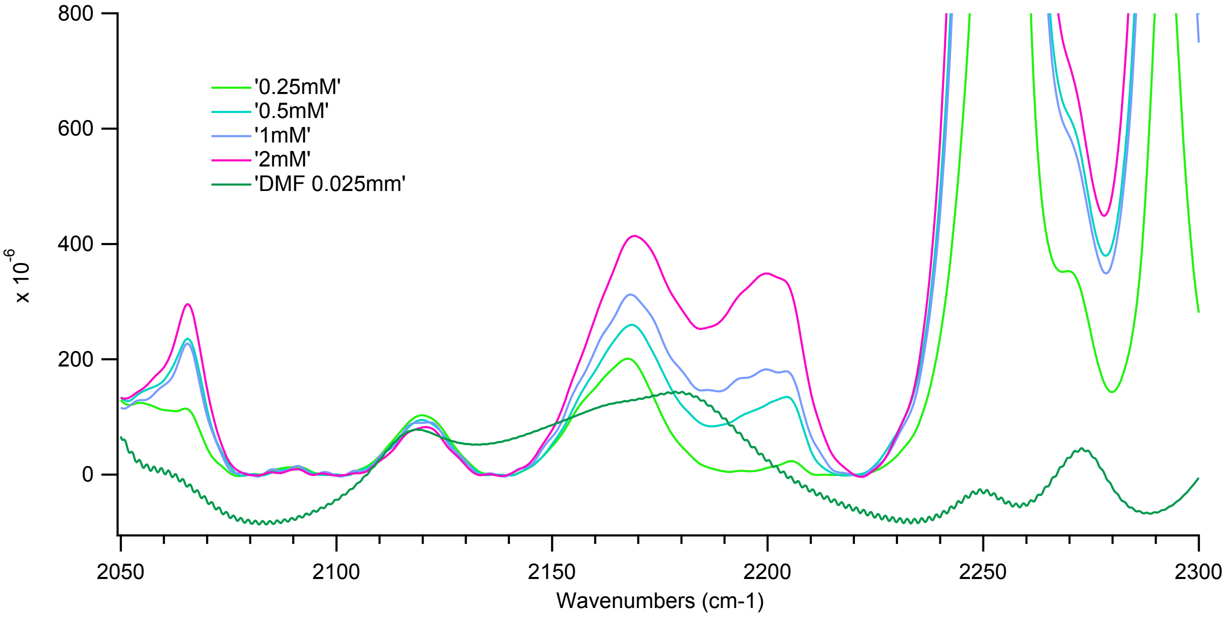

Figure S18: FTIR spectra of alkyne region for PbS/Tc-DA exchanged with varying concentration of Tc-DA compared to neat DMF. While S24 and S25 show that DMF outcompetes or otherwise makes the carboxylate and aliphatic regions difficult to analyze, DMF does not outcompete the peaks at 2168 and 2200 cm^{-1}. The peak at 2120 cm^{-1} matches well to that of neat DMF, but the two alkyne peaks are clearly distinct from DMF's absorbance from 2150-2210 cm^{-1}. Further, the amount of DMF is nearly equivalent between all of the exchanged QD samples, so the trends observed in the two alkyne peaks should be independent of DMF contribution.

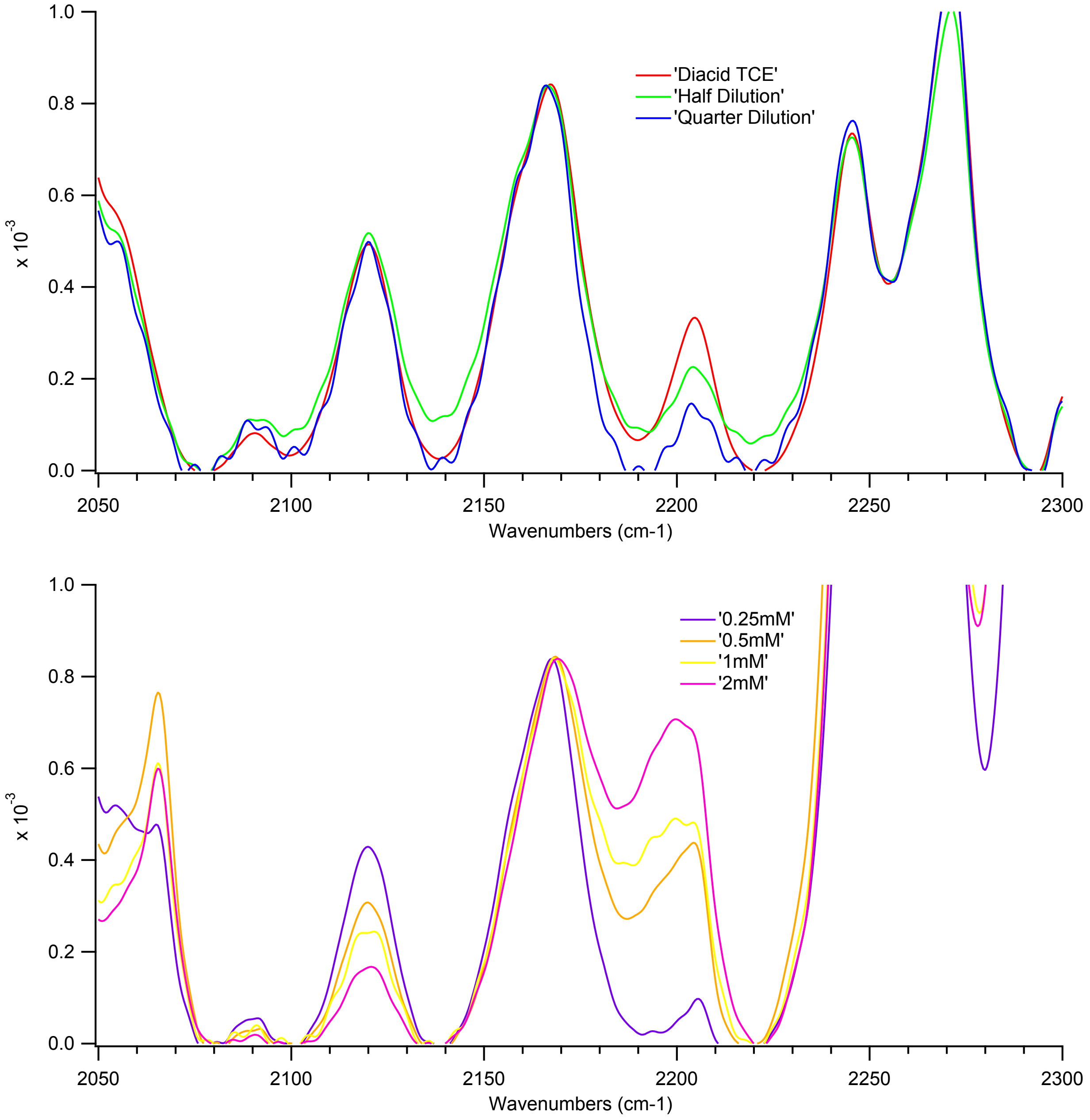

Figure S19: FTIR spectra comparing alkyne region normalized to the peak at 2168 cm^{-1} for neat Tc-DA (top) and solutions of PbS/Tc-DA exchanged with varying concentrations of Tc-DA (bottom). While the 2200 cm^{-1} peak does appear to slightly grow in with increasing concentration for neat Tc-DA, the effect is much less pronounced compared to the Tc-DA exchanged QDs. Additionally, the broadening and shifting for the 2200 cm^{-1} peak with increasing concentration does not occur in neat Tc-DA. This discrepancy provides further evidence towards the growth and broadening of the 2200 cm^{-1} peak in PbS/Tc-DA being associated with a different surface geometry of Tc-DA.

Theory

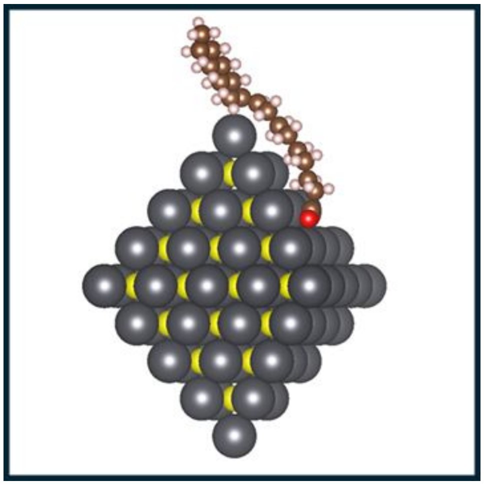

Figure S20: Converged geometry optimization for 2.37 nm PbS/Oleate system.

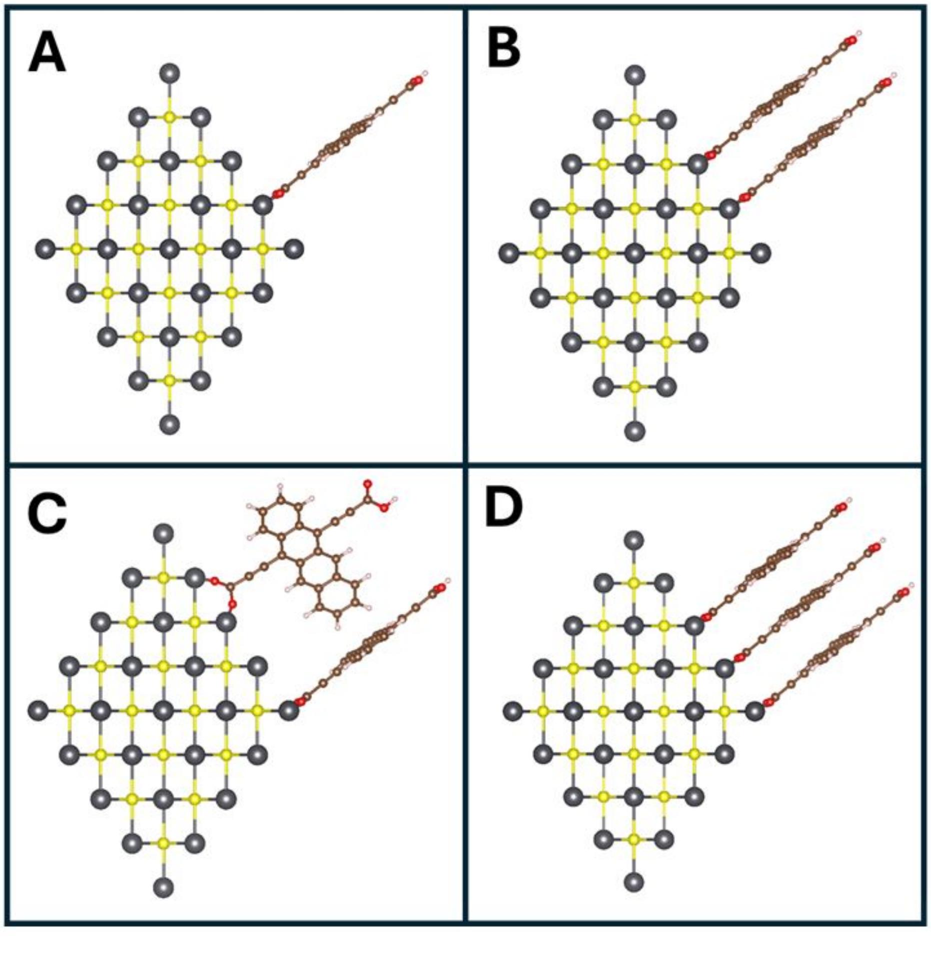

Figure S21: Initial geometries for geometry optimization for 2.37 nm PbS/Tc-DA systems with 1 Tc-DA (A), 2 Tc-DA initialized in parallel (B), 2 Tc-DA initialized orthogonally (C), and 3 Tc-DA (D).

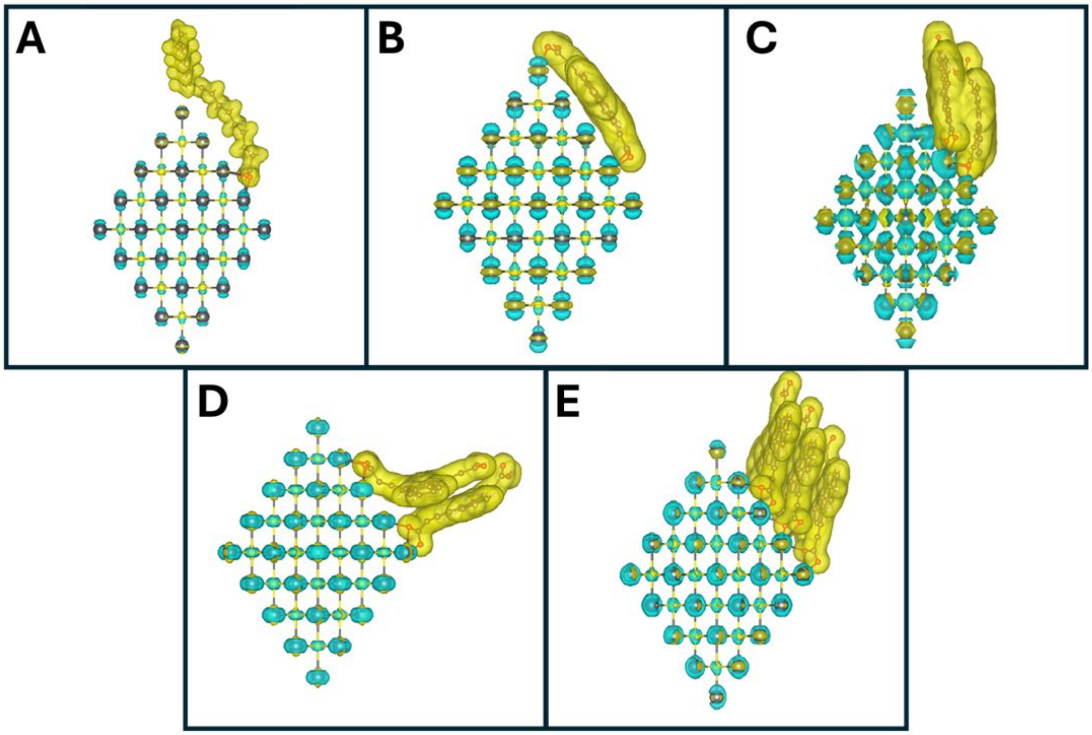

Figure S22: Charge density difference isosurface plots (1e^{-4} e/Å^3 isosurface level) with the isolated PbS QD charge density subtracted highlighting the effect of the ligands on the QD charge density. Results are shown for 2.37 nm PbS/Oleate system (A) and 2.37 nm PbS/Tc-DA systems with 1 Tc-DA (B), 2 Tc-DA initialized in parallel (C), 2 Tc-DA initialized orthogonally (D), and 3 Tc-DA (E).

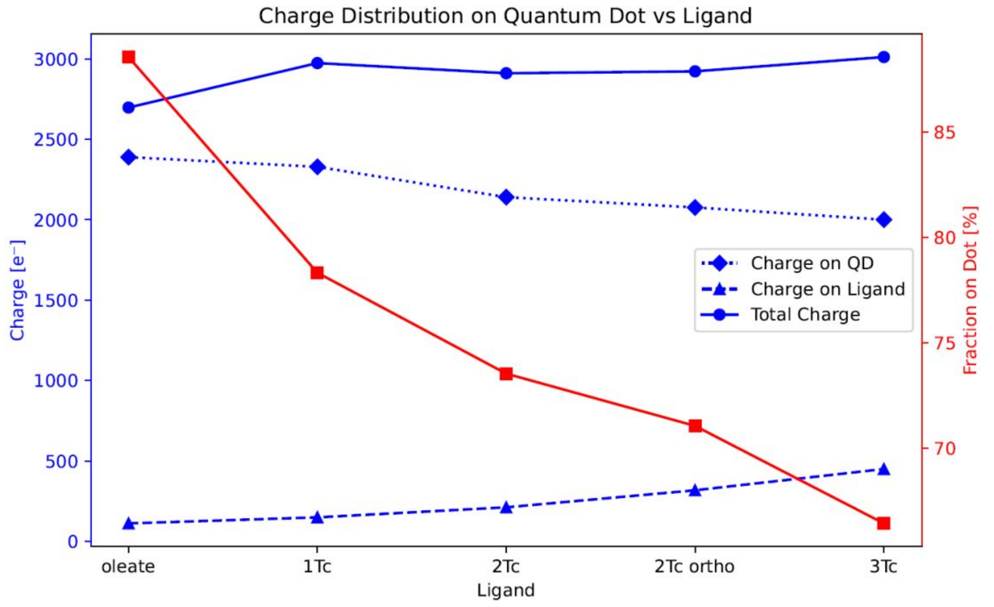

Figure S23: Charge on QD/ligand components for each system obtained from integrating the total charge density. The QD charge density decreases with increasing Tc-DA ligand coverage while ligand charge density increases.

Transient Absorption

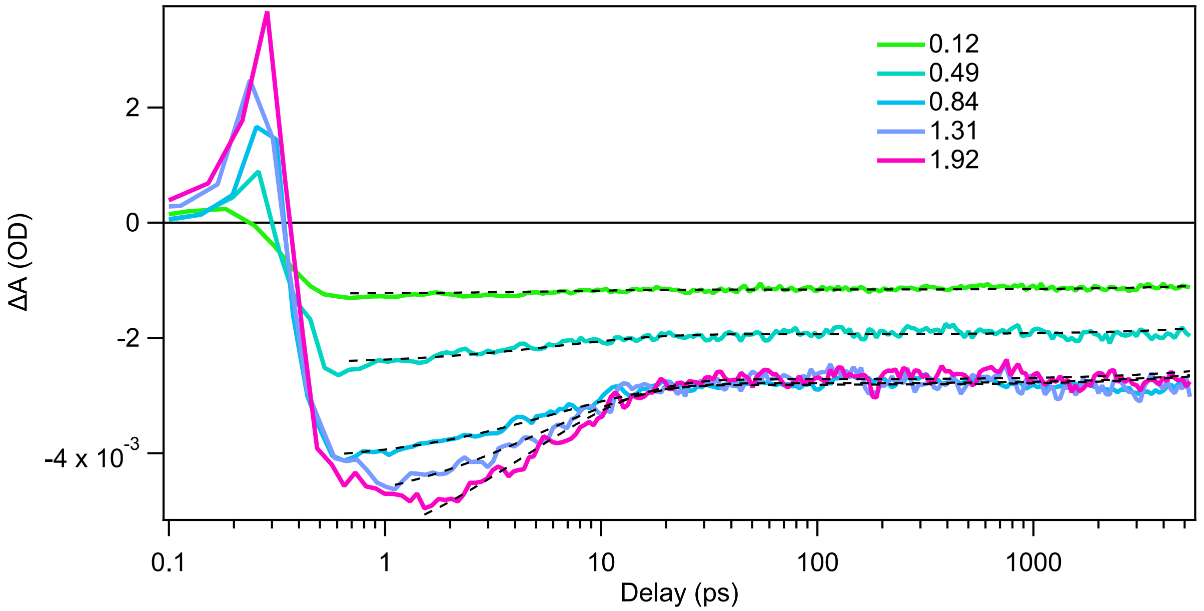

Figure S24: Exciton bleach kinetics for solution of PbS/Oleate with varying fluence. Legend shows <N_0> calculated by global fit to a Poisson biexponential recombination model (dashed lines). Fast decay component (and biexponential contribution) is reduced with lower fluence, confirming the assignment to Auger recombination.

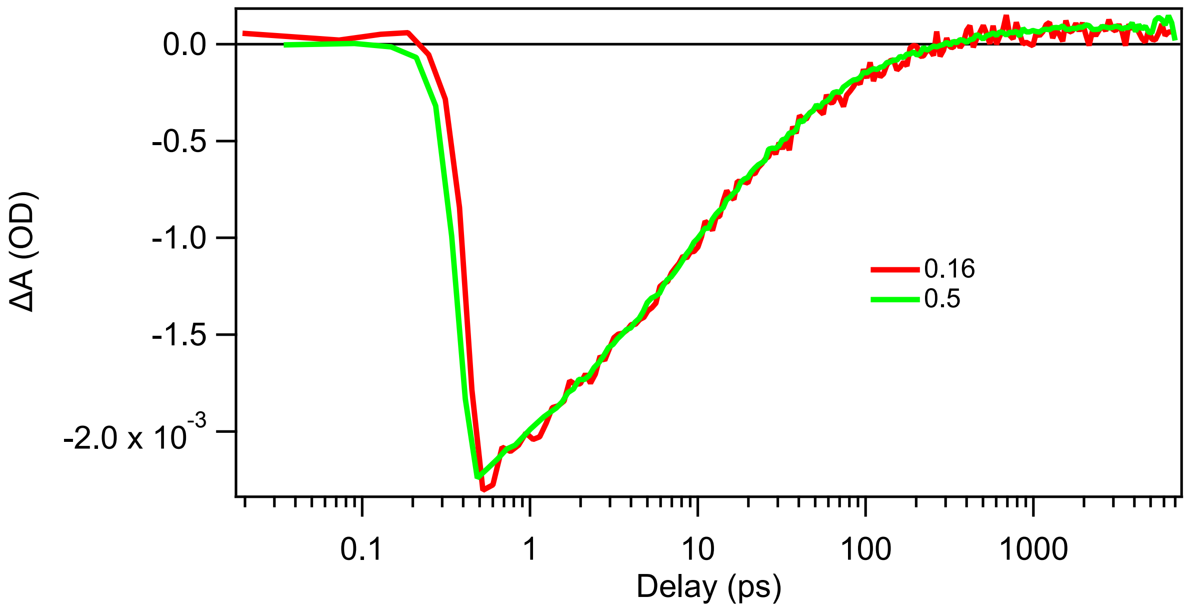

Figure S25: Normalized exciton bleach kinetics for solution of 0.25 mM PbS/Tc-DA with two fluences. Kinetic do not change with increasing fluence, demonstrating that Auger is not significantly contributing.

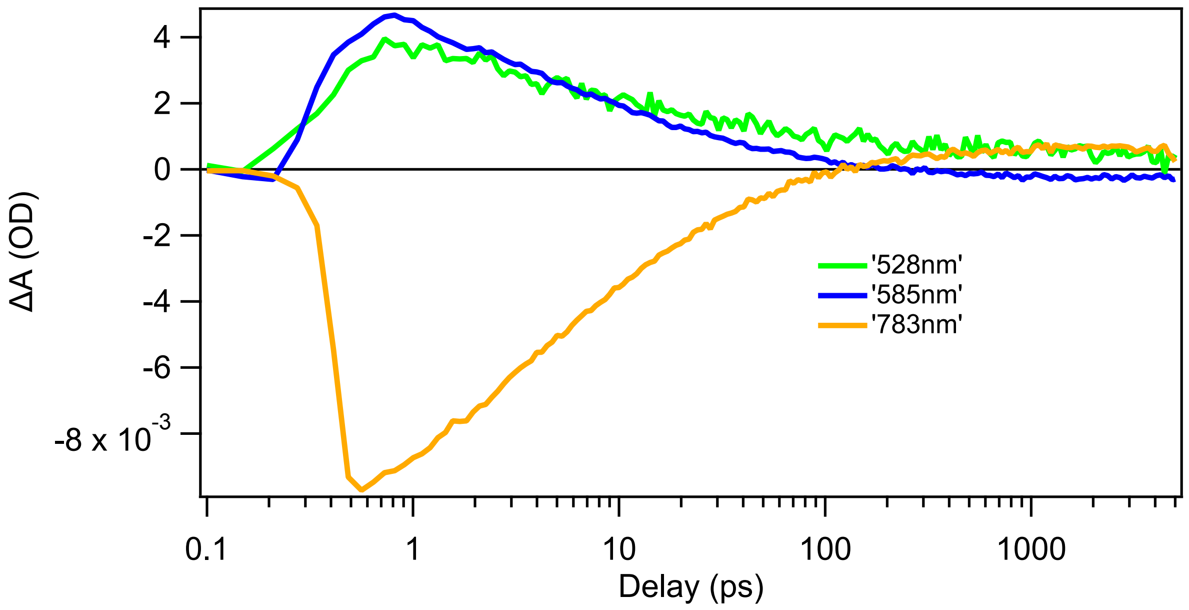

Figure S26: Transient absorption kinetic slices for solution of 0.25 mM PbS/Tc-DA. The orange curve shows quenching of the exciton over ~100 ps. The concurrent growth of the ~580 nm bleach is shown in blue. The growth of the secondary bleach ~540 nm is not present on this timescale for 0.25 mM PbS/Tc-DA, so the green curve remains positive.

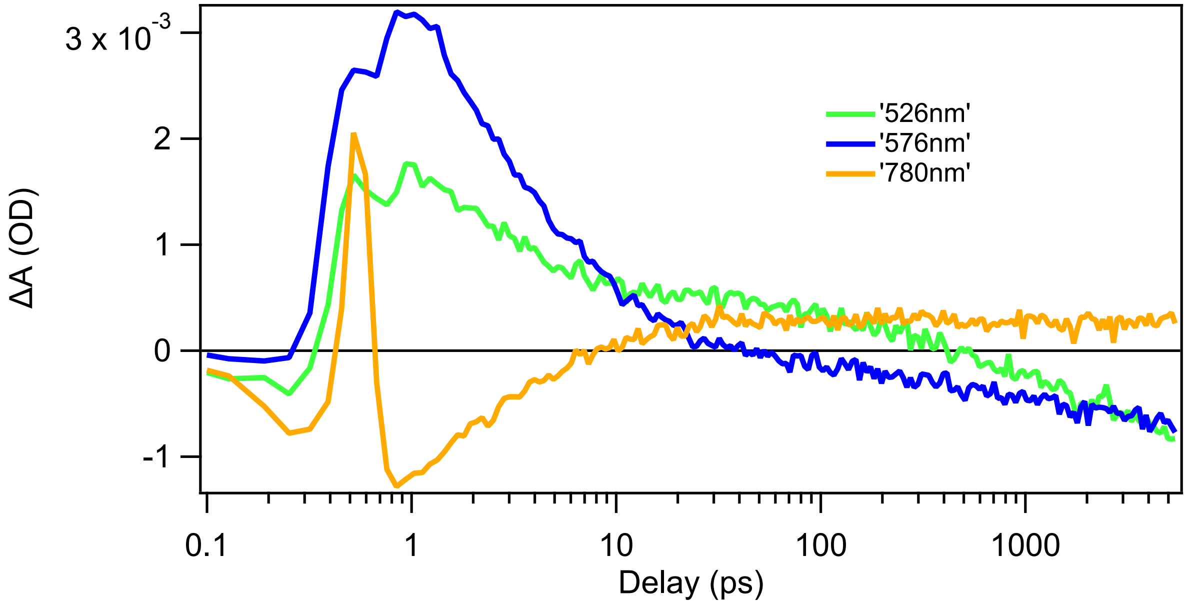

Figure S27: Transient absorption kinetic slices for solution of 1 mM PbS/Tc-DA. Rapid quenching (~10 ps) of the QD exciton is seen in the orange curve. The concurrent growth of the bleach centered ~580 nm is shown in blue. The growth of the second bleach ~540 nm is shown in green. This curve has clear sigmoidal structure demonstrating that multiple mechanisms are in play.

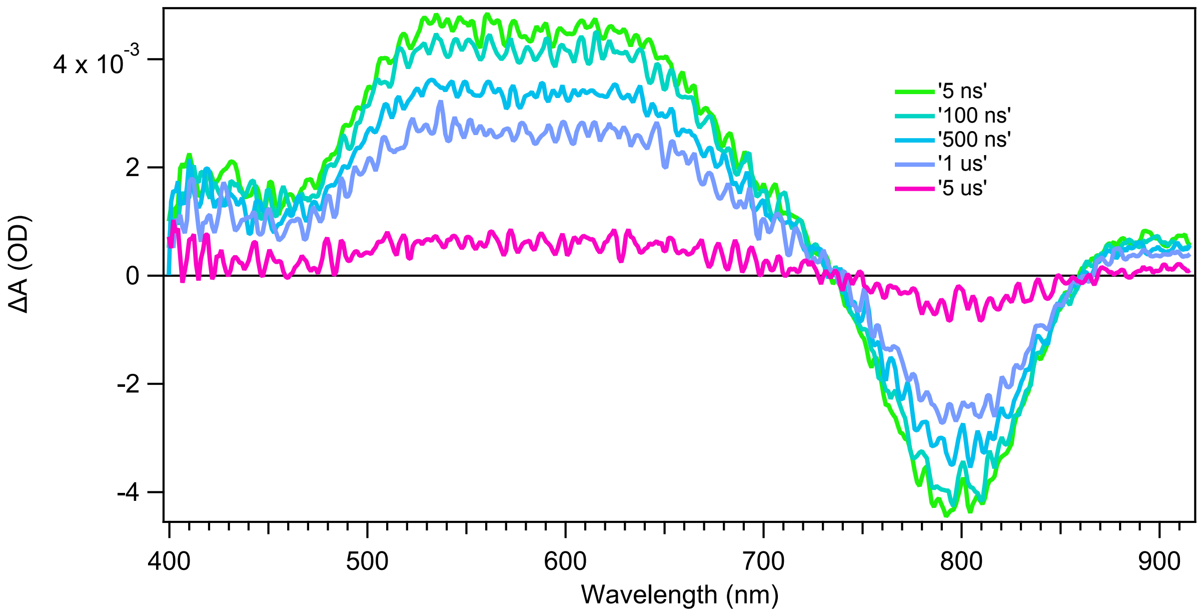

Figure S28: Transient absorption spectral slices from 5ns-5us for solution of PbS/Oleate in TCE.

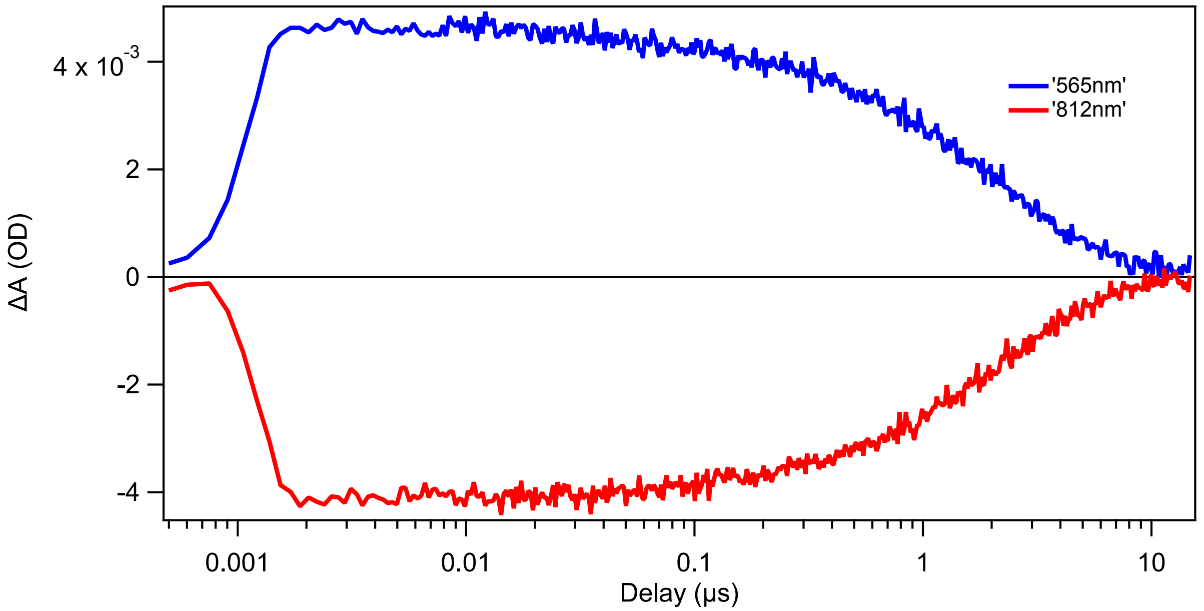

Figure S29: Transient absorption kinetic slices through QD exciton (red) and ESA (blue) for solution of PbS/Oleate in TCE.

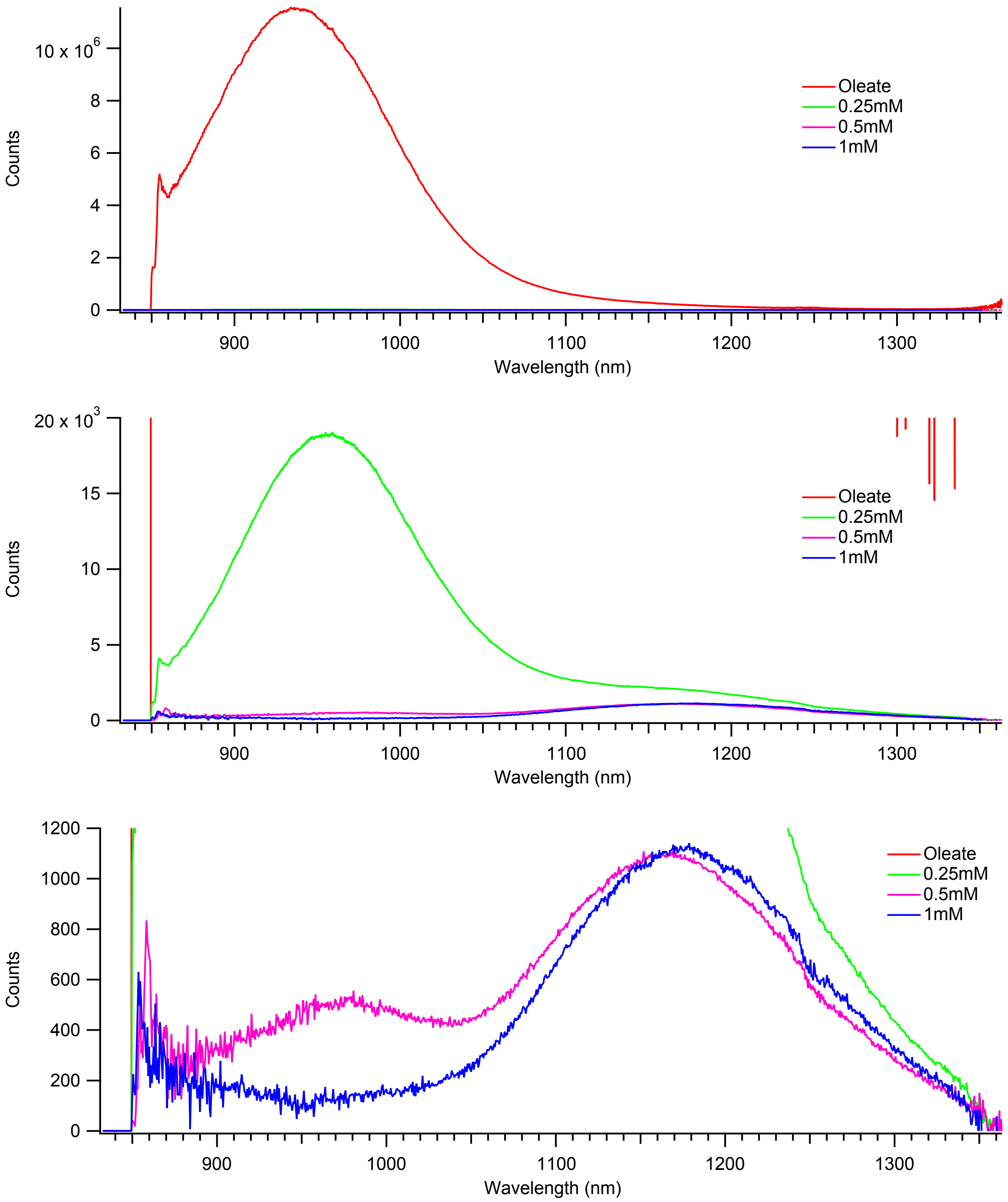

Figure S30: PL spectra adjusted to reflect experiment collection time.

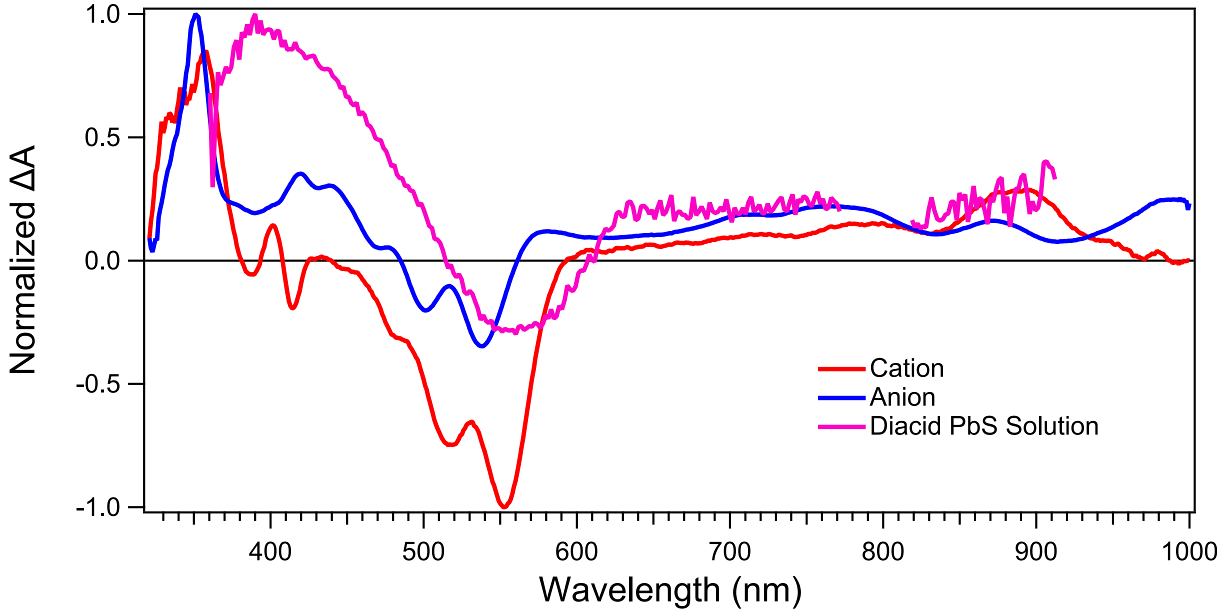

Figure S31: Normalized transient absorption spectra representing the Tc-DA cation (red) and anion (blue) as well as the long-lived feature for a solution of 1 mM PbS/Tc-DA. The cation and anion spectra are taken from Pompetti JACS 2024.[1]

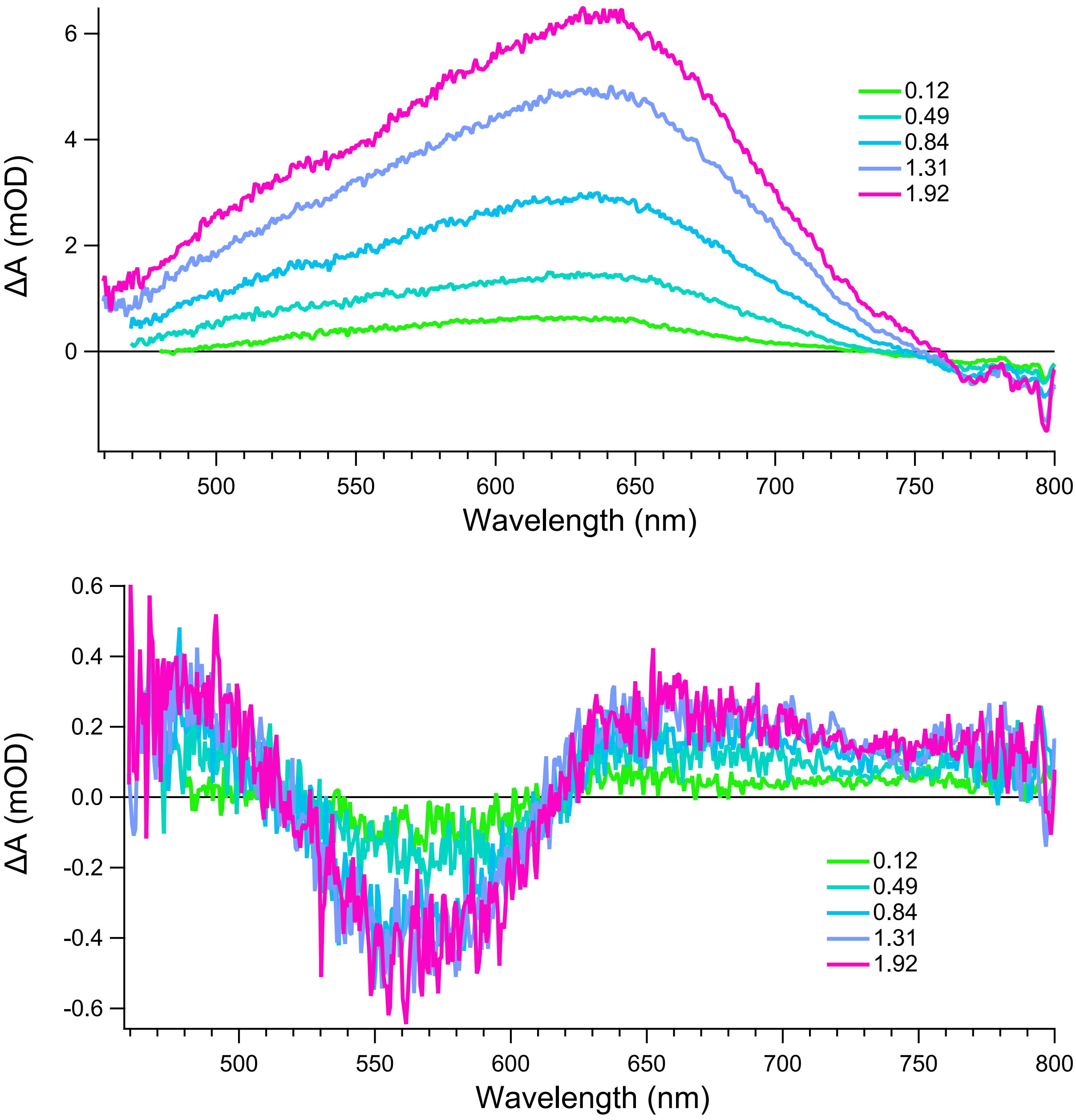

Figure S32: Fluence series transient absorption spectral slices at 1 ps (top) and 5 ns (bottom) for a solution of 1 mM PbS/Tc-DA.

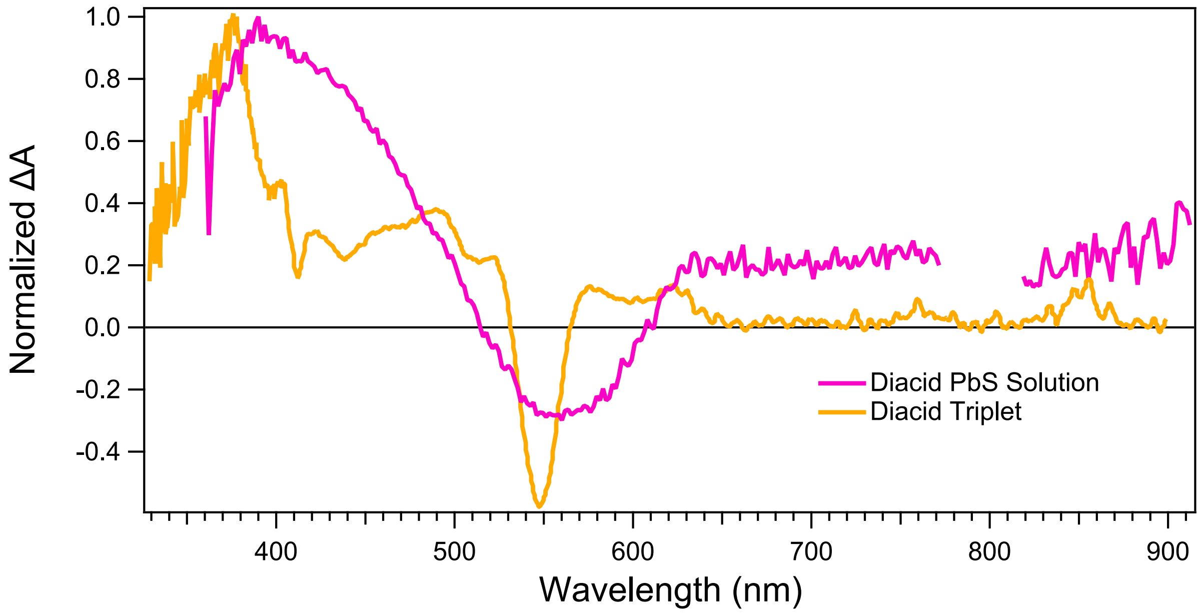

Figure S33: Normalized transient absorption spectra representing the sensitized triplet for Tc-DA and the long-lived feature for a solution of 1 mM PbS/Tc-DA. The sensitized triplet spectrum is reproduced from Pompetti JACS 2024.[1]

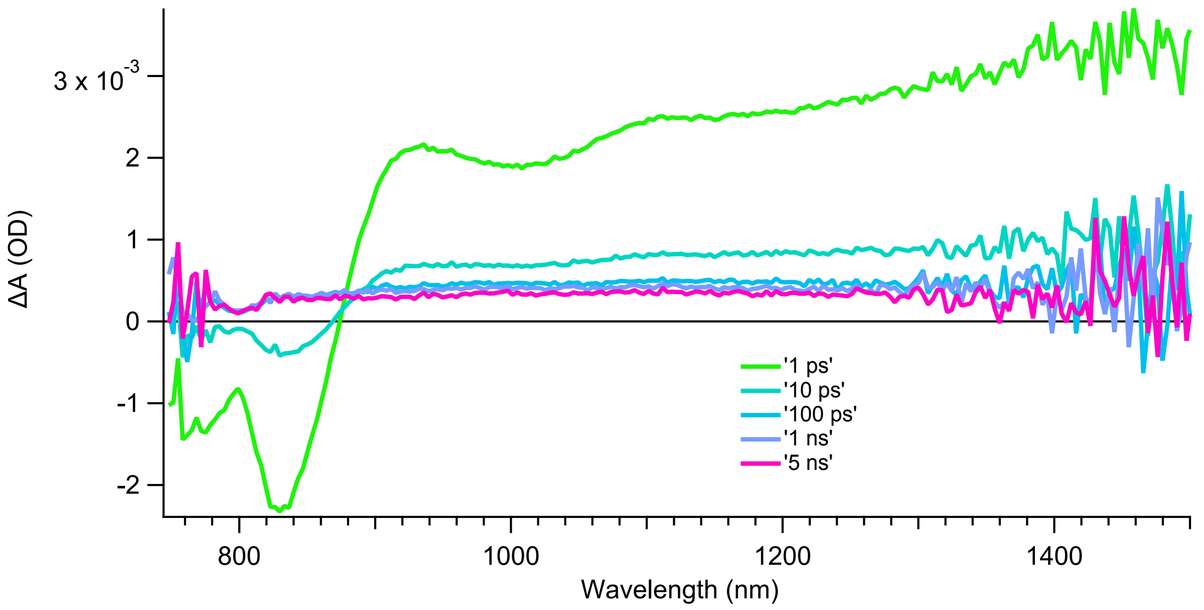

Figure S34: NIR Transient absorption spectral slices from 1ps-5ns for solution of 1 mM PbS/Tc-DA.

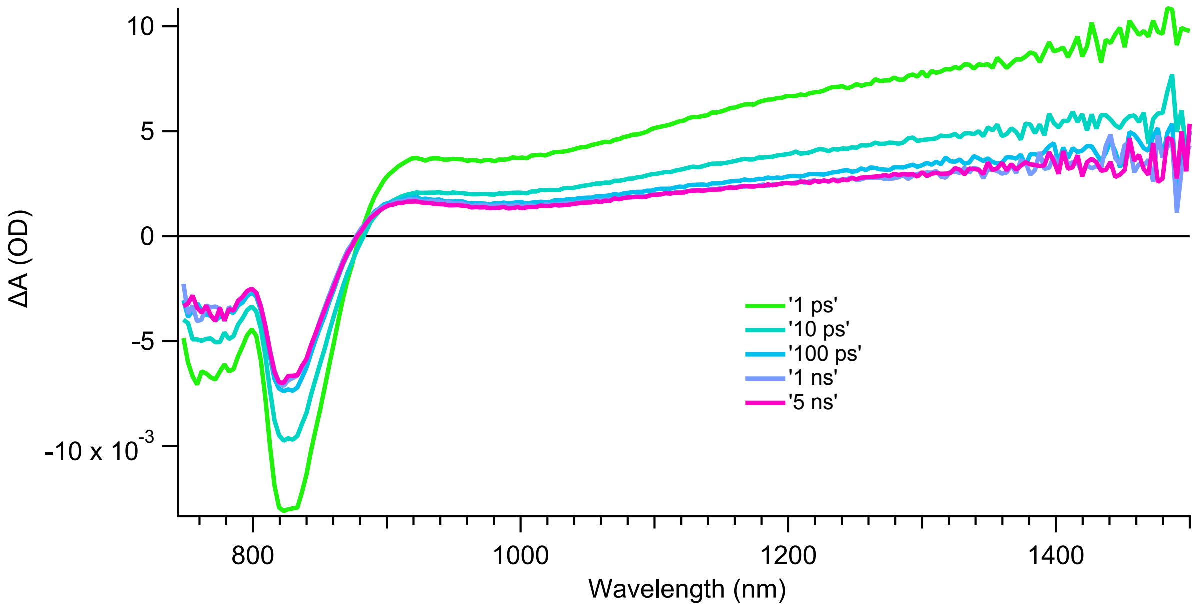

Figure S35: NIR Transient absorption spectral slices from 1ps-5ns for solution of PbS/Oleate.

TA Global Fits

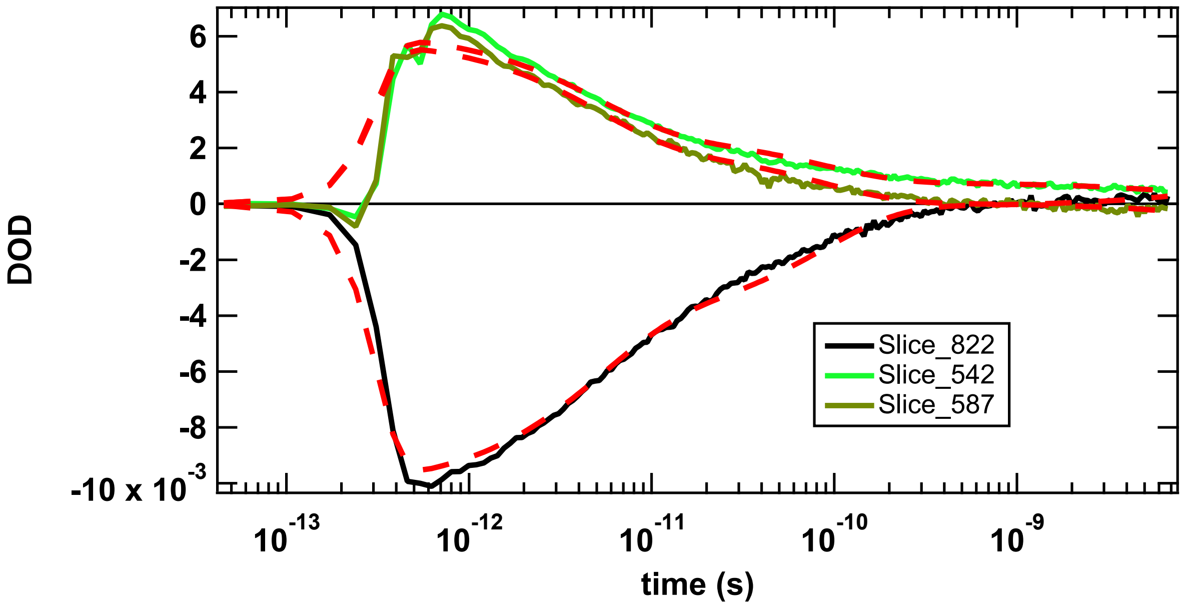

Figure S36: Global fit of fs-ns TA data for 0.25 mM PbS/Tc-DA to $X_{QD} \rightarrow T_{tet} \rightarrow T_{exc} \rightarrow GS$; $X_{QD} \rightarrow T_{hyb} \rightarrow T_{exc} \rightarrow GS$ scheme.

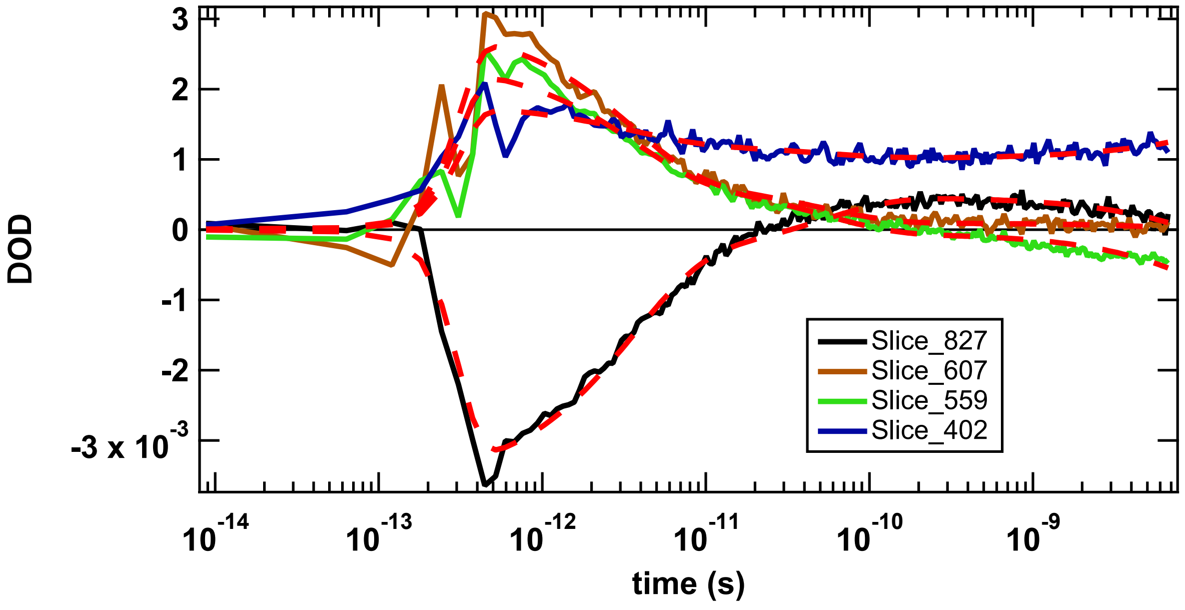

Figure S37: Global fit of fs-ns TA data for 1 mM PbS/Tc-DA to $X_{QD} \rightarrow T_{tet} \rightarrow T_{exc} \rightarrow GS$; $X_{QD} \rightarrow T_{hyb} \rightarrow T_{exc} \rightarrow GS$ scheme.

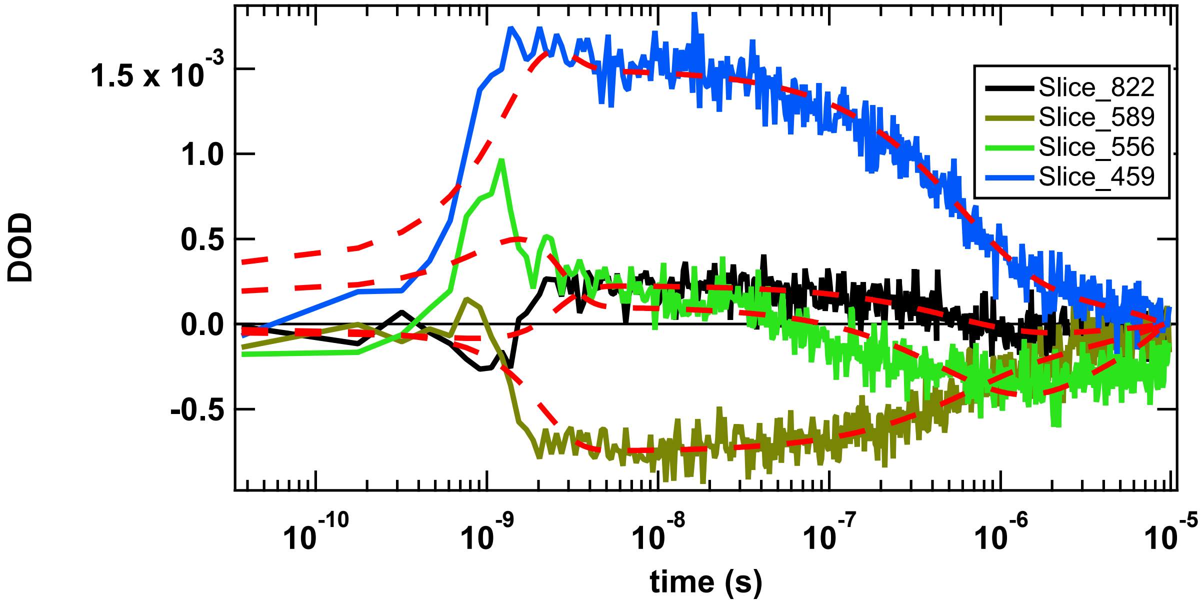

Figure S38: Global fit of ns- μs TA data for 0.25 mM PbS/Tc-DA to $X_{QD} \rightarrow T_{tet} \rightarrow T_{exc} \rightarrow GS$; $X_{QD} \rightarrow T_{hyb} \rightarrow T_{exc} \rightarrow GS$ scheme.

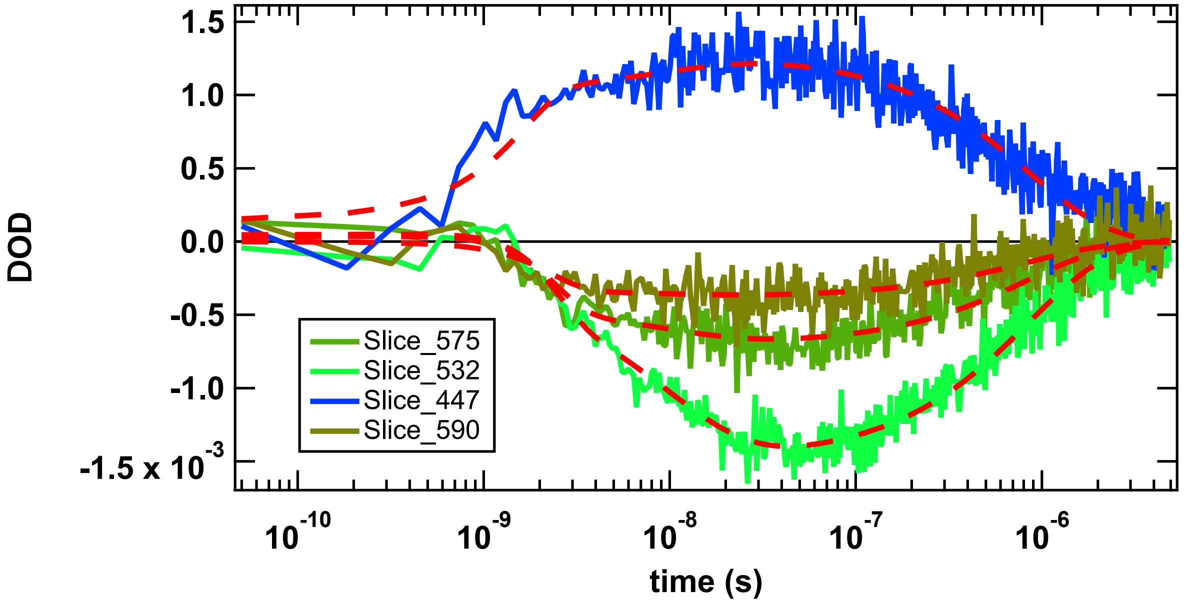

Figure S39: Global fit of ns- μs TA data for 1 mM PbS/Tc-DA to $X_{QD} \rightarrow T_{tet} \rightarrow T_{exc} \rightarrow GS$; $X_{QD} \rightarrow T_{hyb} \rightarrow T_{exc} \rightarrow GS$ scheme.

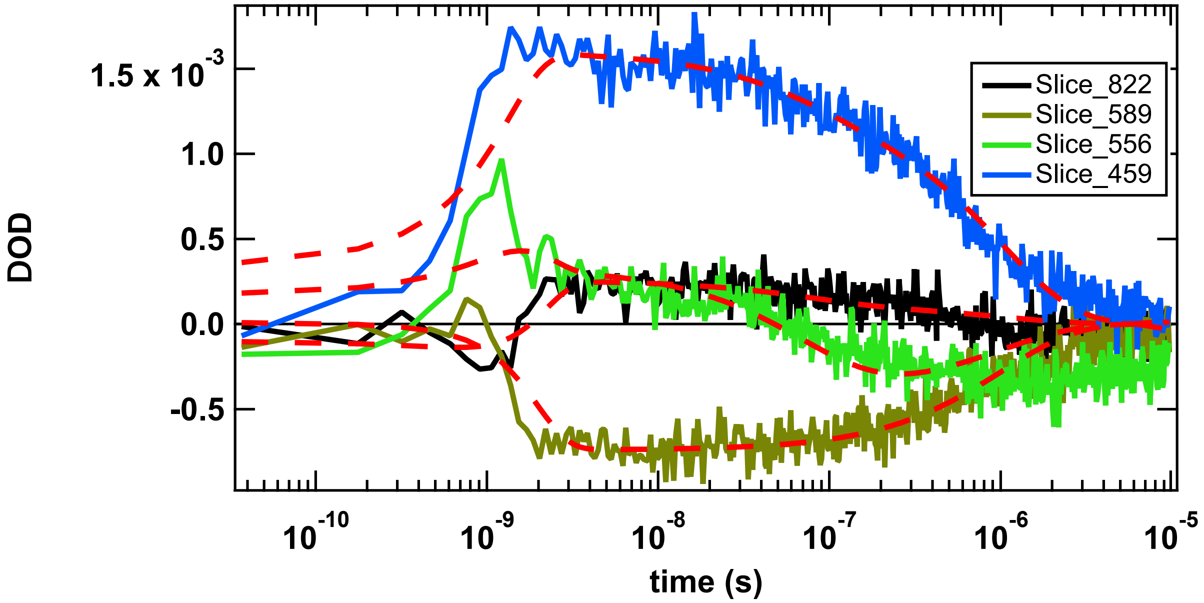

Figure S40: Global fit of ns-μs TA data for 0.25 mM PbS/Tc-DA to sequential model. The sequential model forces faster quenching of the tetracenic triplet species, resulting in a worse fit for the dark and light green curves (589 and 556 nm).

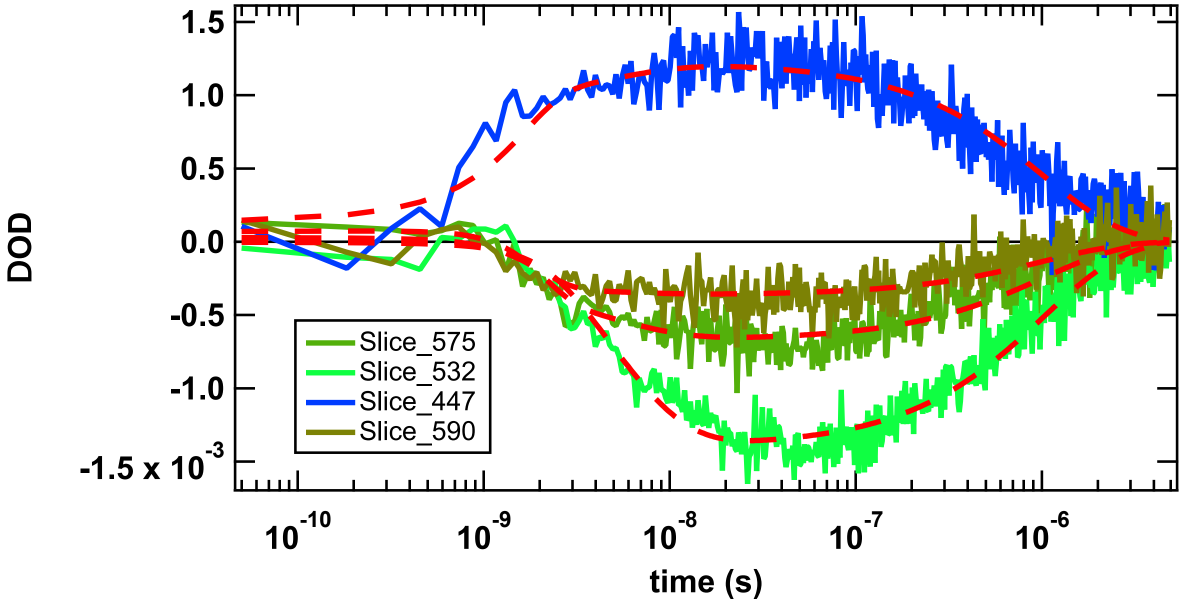

Figure S41: Global fit of ns-μs TA data for 1 mM PbS/Tc-DA to sequential model. Subtly worse fit to light green curve (532 nm) is visible.

References

(1) Pompetti, N. F.; Smyser, K. E.; Feingold, B.; Owens, R.; Lama, B.; Sharma, S.; Damrauer, N. H.; Johnson, J. C. Tetracene Diacid Aggregates for Directing Energy Flow toward Triplet Pairs. *J. Am. Chem. Soc.* **2024**, *146* (16), 11473–11485. https://doi.org/10.1021/jacs.4c02058.

(2) Martinez, M. S.; Nolen, M. A.; Pompetti, N. F.; Richter, L. J.; Farberow, C. A.; Johnson, J. C.; Beard, M. C. Controlling Electronic Coupling of Acene Chromophores on Quantum Dot Surfaces through Variable-Concentration Ligand Exchange. *ACS Nano* **2023**, *17* (15), 14916–14929. https://doi.org/10.1021/acsnano.3c03498.

(3) Deacon, G. B.; Phillips, R. J. Relationships between the Carbon-Oxygen Stretching Frequencies of Carboxylato Complexes and the Type of Carboxylate Coordination. *Coord. Chem. Rev.* **1980**, *33* (3), 227–250. https://doi.org/10.1016/S0010-8545(00)80455-5.

(4) Kennehan, E. R.; Munson, K. T.; Doucette, G. S.; Marshall, A. R.; Beard, M. C.; Asbury, J. B. Dynamic Ligand Surface Chemistry of Excited PbS Quantum Dots. *J. Phys. Chem. Lett.* **2020**, *11* (6), 2291–2297. https://doi.org/10.1021/acs.jpclett.0c00539.

(5) Beygi, H.; Sajjadi, S. A.; Babakhani, A.; Young, J. F.; van Veggel, F. C. J. M. Surface Chemistry of As-Synthesized and Air-Oxidized PbS Quantum Dots. *Appl. Surf. Sci.* **2018**, *457*, 1–10. https://doi.org/10.1016/j.apsusc.2018.06.152.

(6) Sukharevska, N.; Bederak, D.; Goossens, V. M.; Momand, J.; Duim, H.; Dirin, D. N.; Kovalenko, M. V.; Kooi, B. J.; Loi, M. A. Scalable PbS Quantum Dot Solar Cell Production by Blade Coating from Stable Inks. *ACS Appl. Mater. Interfaces* **2021**, *13* (4), 5195–5207. https://doi.org/10.1021/acsami.0c18204.

(7) Auer, B.; Kumar, R.; Schmidt, J. R.; Skinner, J. L. Hydrogen Bonding and Raman, IR, and 2D-IR Spectroscopy of Dilute HOD in Liquid D2O. *Proc. Natl. Acad. Sci.* **2007**, *104* (36), 14215–14220. https://doi.org/10.1073/pnas.0701482104.
